# Amyloid-β oligomers, curvilinear and annular assemblies, imaged by cryo-ET, cryo-EM, and AFM

**DOI:** 10.1126/sciadv.adx9030

**Published:** 2025-08-27

**Authors:** Ruina Liang, Anum Khursheed, Bogachan Tahirbegi, Andrea P. Torres-Flores, Shang Qi, Yao Tian, Hui Zhang, Piotr Szwedziak, Vladimir A. Volkov, Vidya C. Darbari, John H. Viles

**Affiliations:** ^1^ Department of Biochemistry, School of Biological and Behavioural Sciences, Queen Mary University of London, London, UK.; ^2^ College of Life Sciences, South-Central Minzu University, Wuhan 430074, China.; ^3^ Laboratory of Structural Cell Biology, Centre of New Technologies, New University of Warsaw, 02-097 Warsaw, Poland.; ^4^ Center for Microscopy and Image Analysis, University of Zurich, Switzerland.

## Abstract

Prefibrillar structures of the amyloid-β (Aβ) peptide are central to cytotoxicity in Alzheimer’s disease. Time-resolved imaging of oligomers has enabled quantification of their extension. A snapshot of these prefibrillar assemblies has been characterized using a combination of cryo–electron tomography (cryo-ET), cryo–electron microscopy (cryo-EM) single-particle analysis, and atomic force microscopy (AFM). A highly consistent diameter for all curvilinear protofibrils and oligomers of 2.8 nanometers suggests that these assemblies are structural extensions from the smaller oligomers. In situ AFM confirms that spherical oligomers template and extend over time into curvilinear protofibrils. Furthermore, their basic cross section suggests that amyloid fibrils might be initiated by the lateral binding of two curvilinear protofibrils. Cryo-ET/EM single particles also reveal ring-shaped annular assemblies. These have a central internal channel, ~1.4 nanometers in diameter, which is capable of traversing lipid membranes. Large conductance recorded using patch-clamp electrophysiology matches the internal diameter of the Aβ annular architecture.

## Teaser

Four imaging techniques reveal the architecture of prefibrillar amyloids, central to cytotoxicity in Alzheimer’s disease.

## INTRODUCTION

The amyloid-cascade/oligomer hypothesis describes the self-assembly of a 42 amino acid peptide, Amyloid-β (Aβ_42_), and accounts for the most common form of dementia—Alzheimer’s disease (AD) (*1*–*4*). Recent success of therapeutics targeted at Aβ clearance supports the amyloid-cascade/oligomer hypothesis (*5*, *6*). Thus, understanding the self-assembly of monomeric Aβ through oligomeric and curvilinear protofibrils and annular and fibrillar structures is a key question for AD and other protein misfolding diseases (*3*, *4*, *7*, *8*). The process of fibril assembly occurs via a polymerization-nucleation reaction, with an initial lag phase in which nucleating assemblies (oligomers) are formed. This is followed by rapid formation of fibrils that elongate by a templated addition of Aβ to the growing ends (*9*). The prefibrillar assemblies are generally believed to be the cytotoxic form of Aβ_42_ and to be responsible for the cascade of events, which leads to dementia (*10*–*14*). Hence, these important oligomeric assemblies have been the subject of a number of reviews (*3*, *4*, *7*, *15*, *16*). In particular, prefibrillar Aβ_42_ has been shown to disrupt membrane integrity, which causes unregulated cellular Ca^2+^ influx (*17*–*20*), while, in turn, the anionic phospholipid bilayer has been shown to accelerate Aβ fibril formation (*21*).

Aβ amyloid fibrils with a repeating intramolecular cross-β motif have been the subject of numerous structural studies at near atomic resolution (*22*–*28*). However, these fibril structures are not thought to be cytotoxic, although they are able to surface catalyze the formation of toxic oligomers and curvilinear protofibrils (*19*, *29*). Obtaining high resolution structures of the smaller, metastable prefibrillar assemblies is more of a challenge (*7*, *30*, *31*). Biophysical approaches have included nuclear magnetic resonance (NMR) (*7*, *32*–*35*), atomic force microscopy (AFM) (*36*–*40*), and ion mobility mass spectrometry (*41*). Oligomers and curvilinear protofibrils, rather than mature fibrils, insert and carpet lipid membranes (*17*, *18*, *42*); this is believed to induce membrane permeability (*19*, *20*). Annular ring structures composed of Aβ have been suggested to span lipid bilayers and form ion-channel pores that cause cellular Ca^2+^ influx (*43*–*46*). However, these annular pore-like structures remain controversial, as it is not clear, from the AFM (*44*, *47*) and heavy metal negatively stained two-dimensional (2D) transmission electron microscopy (TEM) images (*48*), whether the indentation imaged in these assemblies can traverse the membrane to form a channel pore. Ex vivo, small nonfibrillar assemblies are often referred to as diffusible ligands, water soluble, low molecular weight, and neurotoxic (*11*–*13*, *34*), while annular assemblies have also been isolated from diffuse plaques of the AD brain and imaged by TEM (*49*).

Various prefibrillar assemblies have been described; these include dimers, pentamers, amyloid-derived diffusible ligands, Aβ 56 kDa, Aβ oligomer, curvilinear protofibrils, annular, amylospheroid, and micellar assemblies (*3*, *4*, *7*, *15*). Many of these assemblies have similar properties with similar overlapping molecular sizes, secondary structure, and cytotoxicity. The different descriptions of these structures are often arrived at because of the differing techniques to study the prefibrillar assemblies. Here, we present data that indicate many of these structures are extensions of the same structure. Rationalizing the descriptions of these important cytotoxic structures and their relationship with each other is needed.

Although prefibrillar assemblies have been imaged extensively by AFM and TEM with negative staining, single-particle cryo–electron microscopy (cryo-EM) and cryo–electron tomography (cryo-ET) have yet to be used extensively. Here, we used a combination of cryo-ET, AFM, TEM, and cryo-EM single-particle analysis to obtain 3D images of Aβ assemblies at the nanoscale, under near-native conditions. Using a combination of imaging approaches, we achieved a more holistic understanding of these assemblies. We present evidence to indicate that the smallest of oligomers and curvilinear protofibrils are a continuum of the same structure, and we argue that the lateral self-association of two curvilinear protofibrils forms a basic stable amyloid fibril. We reveal the molecular architecture of the ringlike annular assemblies under near-native conditions, to show how the internal channels relate to Aβ ion-channel pore conductance. The molecular architecture of prefibrillar assemblies has revealed the relationship between different metastable structures and membrane permeability.

## RESULTS

### Imaging lag-phase prefibrillar assemblies

**Fig. 1.**
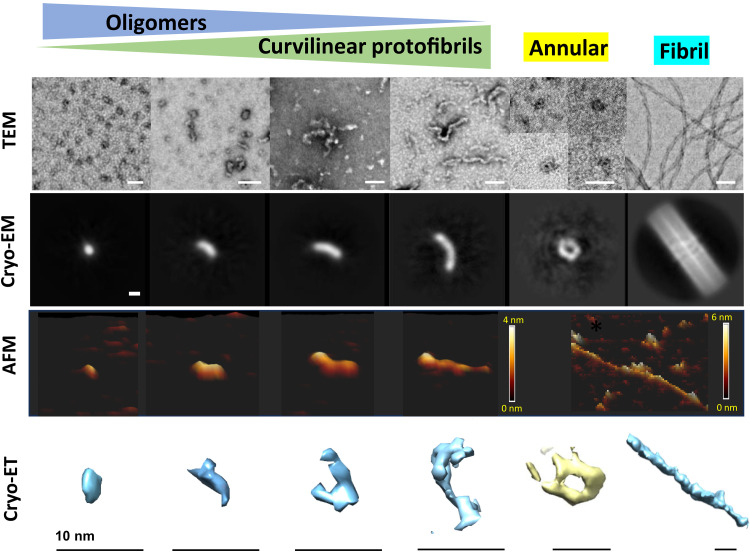
Overview of predominant Aβ_42_ prefibrillar assemblies. Imaged by ns-TEM (first row) (scale bars, 50 nm). Cryo-EM 2D class averages (second row) (scale bar, 2 nm). AFM (third row), shown as 3D surface topology. Oligomer and curvilinear protofibrils are 2.5 to 3 nm in height, while fibrils on the right are 6 nm in height. Cryo-ET (fourth row) 3D tomograms (scale bars, 10 nm).

Assembly of Aβ_42_ monomers results in a number of structures, which coexist as a heterogeneous mixture. Over time, these are replaced by amyloid fibrils. We compared images of the main representative types of assemblies that dominate at the end of the lag phase using four imaging techniques: negatively stained TEM (ns-TEM), AFM, 3D cryo-ET, and 2D class averages from single-particle cryo-EM; see Fig. 1. Structures include small circular oligomers, 2.5 to 2.8 nm in diameter, through to extended curvilinear protofibrils, typically up to 200 nm in length. There are also some larger, more circular structures often 6.5 nm in diameter, which can have an annular (ring) appearance. A limited number of more ordered mature fibrils are also present in lag-phase assemblies, with a 6-nm diameter (Fig. 1). We note that obtaining images of these small structures is a challenge; however, even the smaller oligomers, 2.5- to 2.8-nm diameter, are very different from buffer or Aβ_42_ monomer controls; see fig. S1 for the four different imaging techniques.

**Fig. 2.**
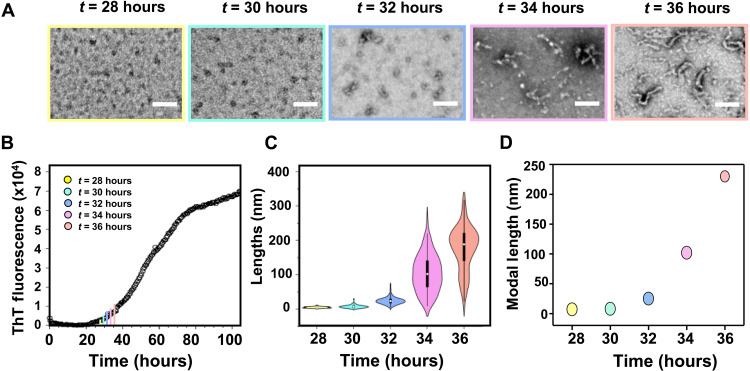
Aβ_42_ oligomer elongation with time. Aβ_42_ prefibrillar assemblies representing five growth time points along with their respective negatively stained TEM images (scale bars, 100 nm). (**A**) Aβ_42_ peptides collected at *t* = 28 hours, *t* = 30 hours, *t* = 32 hours, *t* = 34 hours, and *t* = 36 hours. Typically, *n* > 100 particles were measured per condition. (**B**) The ThT kinetic trace of size exclusion chromatography (SEC)–purified Aβ_42_ (10 μM), incubated with 20 μM ThT in 50 mM Hepes buffer (pH 7.4) at 30°C. (**C**) Distribution of Aβ_42_ prefibrillar assembly lengths at five different time points; white dots represent median value. (**D**) Change in modal length versus Aβ_42_ growth time.

First, we sought to carefully monitor how the distribution of assembly types varies over time as fibrils form. To achieve this, we sampled an Aβ_42_ (10 μM) solution at crucial time points, as Aβ_42_ monomer assembles into oligomers, curvilinear protofibrils, and fibrils. Each time point was imaged by ns-TEM while directly monitoring the total fibril load using the fluorescent dye, thioflavin T (ThT) (Fig. 2 and fig. S2). It is notable that relatively few detectable assemblies are apparent for much of the lag phase. Toward the end of the lag phase (*t* = 28 hours), the micrographs become markedly populated with the smallest of the oligomers, circular in appearance and just a few nanometers in diameter. Within a few hours, the number of extended curvilinear protofibrils increases and dominates. We quantified the distribution of prefibrillar assembly lengths with time. The violin plot shows the distribution of oligomers and curvilinear protofibril lengths at each time point (Fig. 2C). The modal lengths have been plotted versus time (Fig. 2D), increasing from ~5 nm (*t* = 28 hours) to 225 nm (*t* = 36 hours). This steady increase in curvilinear protofibril length (Fig. 2) hints that the oligomers are converting into curvilinear protofibrils directly.

The ThT signal remains relatively low, less than 15% of its maximal fluorescence, even as the number of curvilinear protofibrils observed in the micrographs reaches its maximum at *t* = 36 hours. The lack of the ThT signal confirmed previous studies that suggest prefibrillar structures produced relatively weak ThT signal (*50*). As the ThT signal approaches the 50% maximal point (*t* = 50 hours), the number of curvilinear protofibrils has reduced and is replaced by amyloid fibrils (fig. S2). At the plateau stage (*t* = 84 hours), very few oligomers and protofibrils are apparent, and the micrographs have many long unbranched fibrils present. Oligomers are described as an intermediate to fibril formation, and thus, the steepest part of a fibril growth curve, close to the midpoint of fibril growth, is often assumed to have the most abundant number of oligomers present. Here, we show that prefibrillar assemblies (oligomers or curvilinear protofibrils) reach their maximum abundance, at the end of the lag phase, at just 10 to 15% of maximum ThT signal (Fig. 2 and fig. S2), while at the midpoint of fibril growth, half of the total monomers remain, but the level of oligomers has substantially reduced.

### Curvilinear protofibrils imaged by cryo-ET

Next, we chose to study prefibrillar assemblies via cryo-ET. Cryo-ET has some important advantages as an imaging approach; however, the technique has not been used to study prefibrillar assemblies in detail. In particular, the cryo-tomograms generated produce 3D structures suspended in ice and do not require heavy-metal staining. This improves resolution and reduces artifacts as compared to room-temperature TEM. Furthermore, the technique directly images individual particles in 3D.

The Aβ_42_ preparations (5 μM) have been studied at the end of the lag phase before appreciable fibril formation. These metastable structures rapidly form fibrils, so there was no attempt to isolate or separate prefibrillar assemblies any further; instead, we studied these assemblies as a heterogeneous mixture. In this way, we obtained a “snapshot” of the assembly process, which contains all the assembly forms, from appreciable monomers through to mature amyloid fibrils.

**Fig. 3.**
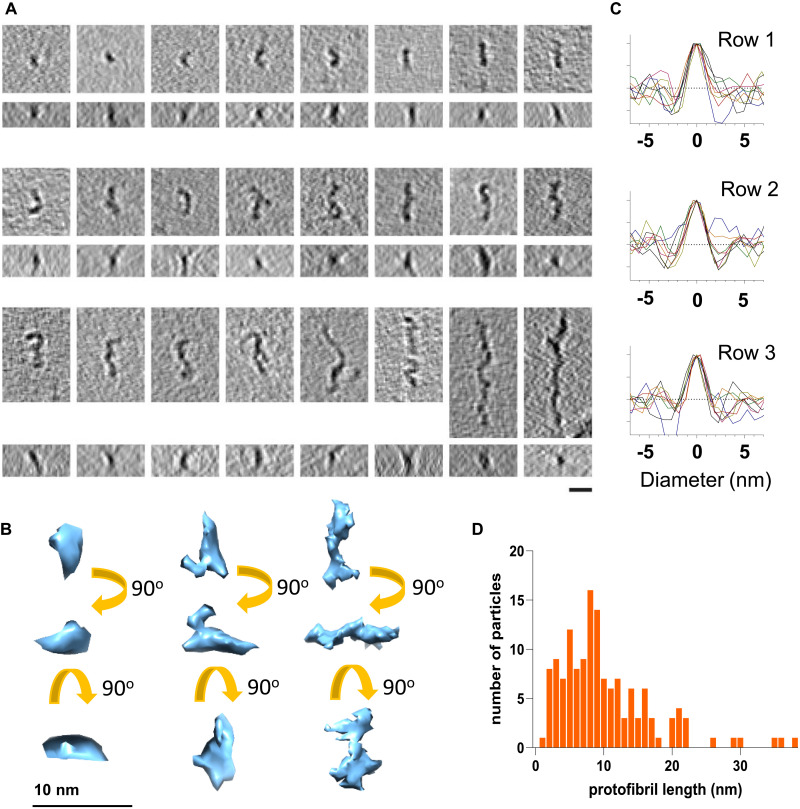
Cryo-ET imaging of lag-phase Aβ_42_ assemblies. (**A**) Cryo-ET 3D tomogram slices of typical oligomers, side and orthogonal top view, are shown (top row), and curvilinear protofibrils (rows 2 and 3). Lengths of the long axis are top row: 5 to 15 nm, second row: 15 to 25 nm, and third row: 25 to 60 nm. Images are presented as sums of 5 to 10 2D slices, each 7.8 Å thick. Scale bar, 10 nm. (**B**) Rendered examples of 3D tomographic images of oligomers and curvilinear protofibrils. (**C**) Density profiles, orthogonal to the long axis of the protofibril; eight traces are shown in each row for each structure. A consistent diameter of 2.8 nm is apparent. (**D**) Distribution of length (in nanometers) for individual protofibril particles; most curvilinear protofibrils are less than 20 nm in length but consistently ~2.8 nm in diameter at this point in the lag-phase assembly. Aβ_42_ (5 μM monomer equivalent).

Representative 3D tomogram slices of prefibrillar structures are shown in Fig. 3A. These structures have been arranged in increasing length. The top row contains oligomers with their longest length of 5 to 15 nm, and the middle row shows curvilinear protofibrils between 15 and 25 nm long, while the final row contains single particles, 25 to 60 nm in length. The range of lengths identified in the tomograms is mostly less than 30 nm. The distribution of lengths of these oligomers and protofibrils is shown in Fig. 3D. The 3D-rendered surface of typical oligomers and curvilinear assemblies is shown in Fig. 3B; these 3D images are of single particles, and the irregular appearance of the 3D-rendered structures reflects the bends and turns rather than a specific substructure. Branching and pronounced bends in the curvilinear protofibrils are also apparent in the cryo-ET tomograms; examples of which are shown in fig. S3.

A key observation for these data is that all structures presented in Fig. 3C have a very consistent cross section, with a diameter of ~2.8 nm. The very consistent diameters, for the oligomers and curvilinear protofibrils, suggest that they are derived from the same basic fold, with increasing length due to templated Aβ-monomer addition. We believe that this is an important observation, suggesting that assemblies typically described as oligomers (top row) or curvilinear protofibrils (remaining rows) are the same basic structures with increasing linear addition of Aβ molecules.

These oligomers and curvilinear protofibrils have previously been imaged using ns-TEM; however, the heavy-metal staining has caused their diameters to be poorly determined and overestimated; for example, 6 nm is often reported (*51*), rather than 2.8 nm shown in Fig. 3. AFM imaging has previously reported a height of 3 nm but has also overestimated the curvilinear protofibril widths (*36*, *38*–*40*). Occasionally, curvilinear protofibrils are described as “beads on a string,” implying a repeating subunit containing multiple Aβ molecules. However, experimental evidence for this is limited. We see no evidence of a regular undulating structure in our images (Fig. 1).

### In situ, temporal AFM directly shows extension of oligomers to form curvilinear protofibrils

**Fig. 4.**
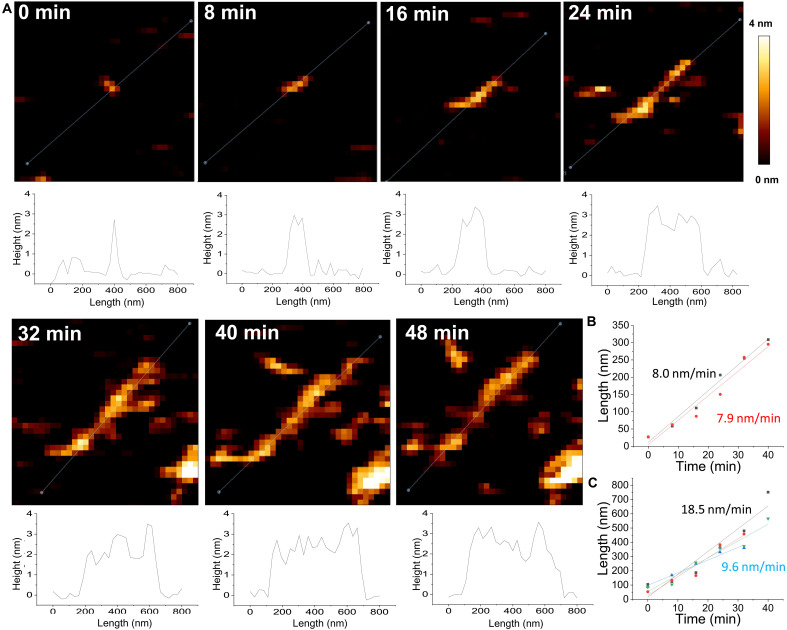
Real-time bidirectional extension of oligomer to form curvilinear protofibrils. (**A**) AFM images on mica support in Aβ_42_ solution (5 μM). Images recorded every 8 min. Height of oligomers and extending curvilinear protofibrils remains typically close to 2.8 nm. Cross-sectional height shown for each image as dashed line. The length of the protofibril has been plotted versus time. Each micrograph is 783 nm by 783 nm with a height of 0.0 to 4.0 nm. (**B**) Linear best-fit line indicates a rate of oligomer extension from each end of 7.9 and 8.0 nm min^−1^. (**C**) Further examples of the rate of different oligomer extension range between 18.5 and 9.6 nm min^−1^. Aβ_42_ (5 μM) growth on mica support at 22°C, pH 7.4, in phosphate-buffered saline (PBS) buffer.

We were very interested in the relationship between the small oligomers and curvilinear protofibrils. The identical diameter of 2.8 nm for the two structural forms suggests a direct relationship; however, our cryo-ET and EM data, frozen in ice, are not able to show this growth and extension directly. For this, we used AFM. We have been able to monitor in real time the temporal assembly of oligomers on a mica surface from a solution of monomeric Aβ_42_ (5 μM), which was deposited on a mica support. A 10 μm–by–10 μm surface was imaged in the Aβ_42_ solution using contact-mode AFM; in this way, we were able to generate an image every 8 min. The first scan (*t* = 0 min) showed a relatively flat mica surface, which was largely indistinguishable from buffer solution, and the next scan (*t* = 8 min) had a few very small assemblies, only a single pixel in length with a 2.5- to 3.0-nm height. Once the oligomer is present on the mica surface, subsequent AFM images show that the oligomers do extend in length. The extension occurs from opposite ends of the oligomers, at a similar rate, indicating a specific bidirectional growth. A typical oligomer elongating to form curvilinear protofibrils is shown in Fig. 4, and six time points are shown in the same position, with 8-min intervals. A further example of oligomer extension is shown in fig. S4. The cross-sectional heights show that the oligomers or curvilinear protofibrils maintain a 2.5- to 3.0-nm height, over the 48-min time period. These structures do not have the appearance of fibrils, which are relatively straight with a height of 5.5 to 6.0 nm (fig. S5). The height of the fibril structure, according to AFM, is in close agreement with the cryo-EM structures of fibrils (*24*). As expected, unlike the curvilinear protofibrils, the 6-nm-high fibrils exhibit some regular periodicity, reflecting their helical twists.

The initial short oligomers that act as nucleation sites for extension to form curvilinear protofibrils are not an unorganized surface for curvilinear protofibrils to grow from. Oligomer extensions are consistently bidirectional, from opposite ends, rather than multiple extensions. This is strongly supportive of a process of templating extension, presumably with a cross-β motif. It is well established that oligomers and curvilinear protofibrils are rich in β sheet structure (*7*, *32*). The rate of extension of the oligomers has been plotted (Fig. 4), and the spherical oligomer (2.5- to 3.0-nm height) can extend to 600 nm length within 48 min. Images recorded beyond this time point lose their resolution, as the AFM probe collects protein aggregates on its tip; in addition, the mica becomes crowded with many assemblies. The rate of extension has been plotted, and this oligomer grows from each end at 8 nm/min (Fig. 4). The growth rate of other oligomers is similar (Fig. 4C and fig. S4). There is no significant difference in the growth rates from either end of the oligomer. The growth and extension of oligomers have a linear growth rate (Fig. 4C). This suggests that the process is a simple monomer addition on the ends of a growing oligomer, with the interface remaining unchanged as the oligomers extend. The rate of this process will be influenced by Aβ monomer concentration near the mica surface, diffusion to the end of the protofibrils, and structural rearrangement to bind and form a cross-β strand. If we assume that each monomer, which adds to the end of a curvilinear protofibril, will increase the length of the protofibril by a single β strand, 0.48 nm, then we can calculate an elongation rate under the conditions used for this AFM experiment. This assumption is based on the dimensions of the protofibril and its relationship to fibril structures (discussed later). This indicates that the oligomer (shown in Fig. 4) has ~16 Aβ molecules adding per minute from a single growing end, under these conditions at the mica surface.

Inspection of the AFM micrographs indicates that not all small oligomers with a ~3-nm diameter exhibit elongation over time. Many of the assemblies remain unchanged over time, and these small assemblies may be amorphous and not able to present a templating cross-β sheet surface.

In situ, real-time AFM imaging of mature amyloid fibril growth has previously been described for Aβ (*52*, *53*), but oligomers or curvilinear protofibril growth was not described. Aβ fibrils also exhibit bidirectional linear growth (*52*). There has been a report of stop/start growth when the growing ends of a fibril become temporarily frozen (*53*).

### Single-particle cryo-EM: A quantitative snapshot of prefibrillar assemblies

Cryo-EM single-particle analysis has also been used to characterize prefibrillar Aβ_42_ assemblies. We used this approach to image and characterize a very large number of particles, with a view to quantifying the range of assembly categories observed at the lag phase of fibril formation. Approximately 3 million particles were picked using “cryolo” autopicking; a typical micrograph is shown in fig. S6. Following the removal of fibrils and artifacts, 2.5 million particles remained. A range of prefibrillar assemblies were apparent from rounds of 2D classification; these are summarized in Fig. 1 and compared with lag-phase assemblies imaged by cryo-ET, AFM, and negatively stained particles imaged by TEM.

**Fig. 5.**
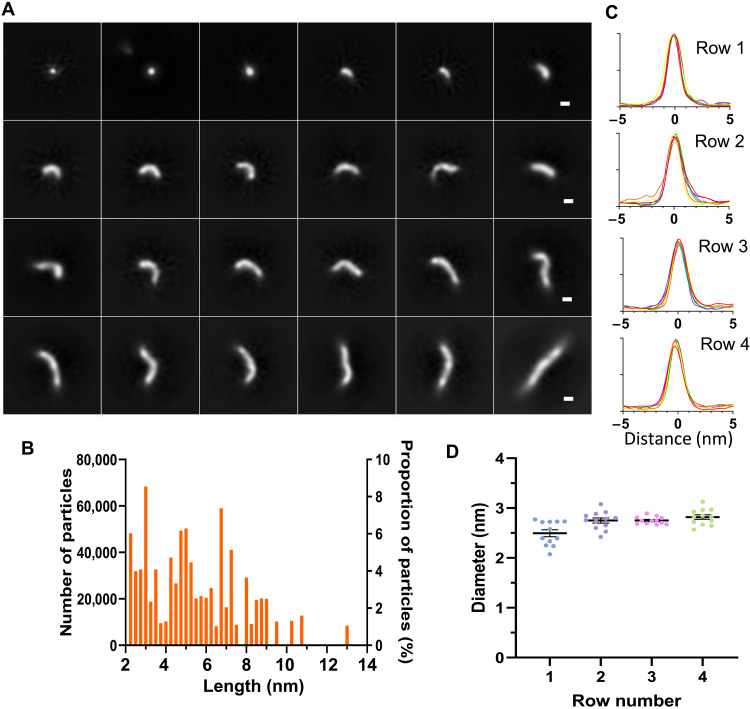
Cryo-EM 2D class averages of typical oligomers and curvilinear protofibrils. A total of 209,000 particles (scale bars, 2.0 nm). (**A**) The class averages have been arranged in increasing length. Top row: 2.5 to 5.0 nm, second row: 5.0 to 8.0 nm, third row: 8.0 to 10 nm, and bottom row: 10 to 14 nm. The cryo-EM class averages have a close resemblance to single particles imaged by the cryo-ET. (**B**) Distribution of oligomer and curvilinear protofibril lengths. Taken from half a million particles, 95% of particles are less than 9 nm long. (**C**) Density profiles of the protofibril diameter. Six profiles for each row (red, orange, and yellow on the left side; green, blue, and purple on the right side). (**D**) Diameters observed for each row. The mean diameter for class averages in rows 2, 3, and 4 is consistently 2.8 ± 0.1 nm (SD).

Most notable from these data was the consistency in the width of most images generated from 2D class averaging. Approximately 80% of the 2.5 million particles in the micrographs produced class averages with a width of 2.8 nm but of varying length and curvature. Representative 2D class averages are shown in Fig. 5. A density plot, orthogonal to the long axis, is also shown for each 2D class average (Fig. 5C). These plots indicate notably consistent diameters of 2.8 ± 0.1 nm (SD). The diameters were measured at 15% above the baseline (Fig. 5D). Some of the smaller globular oligomers shown on the top row have diameters of 2.5 nm by 2.8 nm in the longer axis. The diameters are consistent with measurements of curvilinear protofibrils observed in the cryo-ET tomograms (Fig. 3), as well as curvilinear protofibrils we have directly measured extending in real time by AFM (Fig. 4). The small size of these assemblies is challenging; however, the observations that all 2D class averages have identical 2.8-nm diameter indicate that these particles are not random artifacts. Furthermore, individual oligomers are identifiable in the micrographs (figs. S1 and S6).

A plot of the length versus number of curvilinear protofibrils is shown in Fig. 5B. On average, the actual length of these assemblies is underestimated a little because of the range of possible orientations for each particle. There are a few protofibrils that exceed 200 nm in length, imaged by cryo-EM, cryo-ET, and ns-TEM, and we suggest that this is because longer protofibrils are prone to mechanical shearing during rotational diffusion.

Single particle imaged by cryo-ET (Fig. 3) indicates that these curvilinear protofibrils are extremely variable in curvature, and so class averaging of these particles should be interpreted with caution, particularly as the curvilinear assemblies extend. However, comparisons of these structures imaged by cryo-ET and AFM suggest that this approach has utility, particularly for the shorter structures.

With a snapshot of different assemblies, we performed 3D reconstruction. Using Cryo-SPARC, we performed ab initio reconstruction with all 2.5 million particles to generate 10 heterogeneous 3D reconstructions. These represent various intermediates in the assembly process. The range of assemblies present in the micrographs was sufficiently different to produce distinct molecular architectures. Figure S7 summarizes the appearance of the 10 structures, the number of particles, and typical 2D class averages. Inspection of the 10 ab initio structures (fig. S7) indicated that as many as seven of these structures appear as curvilinear protofibrils. Similar to the 2D class averages, the reconstructed 3D structures contain a consistent diameter of 2.8 nm and show a close resemblance to the 3D structures imaged by cryo-ET (Figs. 1 and 3).

We had hoped the 3D image reconstruction, particularly of the shorter curvilinear protofibrils, might be of sufficient resolution to reveal secondary structure. However, these structures do not have a regular twist as observed for fibrils, and so helical reconstruction used to obtain atomic details of amyloid fibrils is not possible for these curvilinear protofibrils. Combined with their small size, heterogeneity, and presumed inherent flexibility, 3D reconstructions do not reveal atomic details. However, the resolution obtained using cryo-ET and cryo-EM, without need for heavy-metal staining, is an important step forward. The dimensions of these 3D reconstructions indicate an approximate Aβ stoichiometry and hint at the structure relationship with fibrils as discussed in the next section.

### The relationship between curvilinear protofibrils, fibril structures, and annular assemblies

**Fig. 6.**
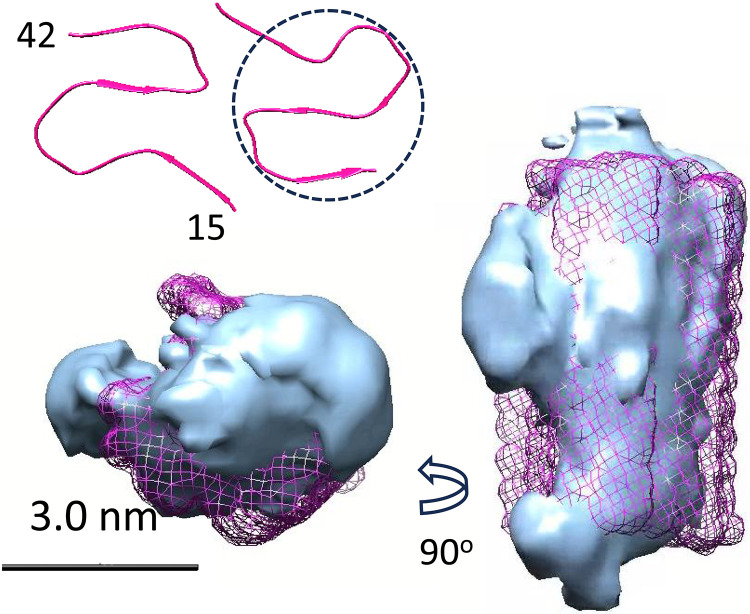
Curvilinear protofibril overlaid with half a fibril. Overlay of curvilinear protofibrils (cyan) with mesh for half a fibril (magenta), and 3D reconstruction from 59,000 single particles. Fibril structure (PDB: 2MXU). Only residues 15 to 42 are shown in fibril (as a mesh), and residues 1 to 14 are often flexible in fibrils. Top left is a cross section of the whole fibril, and top view contains two Aβ molecules with conserved S-shaped fold, showing β strands (PDB: 5KK3). Circle has a diameter of 2.8 nm similar to the curvilinear protofibrils. Scale bar, 3.0 nm.

We were struck by the observation that the cross section of the oligomers and curvilinear protofibrils closely matches the density of half the cross section of the basic Aβ_42_ amyloid fibril. The cryo-EM 3D reconstruction of a curvilinear protofibril, taken from 50,000 particles, is overlaid with half a basic fibril structure [Protein Data Bank (PDB): 2MXU] (Fig. 6) (*23*). Numerous Aβ_42_ fibril morphologies are reported under different conditions, both in vitro and ex vivo. No two structures reported are identical, but all typically contain a well-conserved “S-shaped” topology, between residues 15 to 42 (Fig. 6) (*22*–*24*). We believe that the N-terminal third, residues 1 to 14, is likely to be too disordered to be imaged in the curvilinear protofibrils; even in the more ordered fibrils, these residues are often unresolved (*23*). These residues have been shown to be dynamic and lack stable hydrogen bonds in mature fibrils (*54*). Our 3D reconstructions, which match our cryo-ET data, should be fitted to the molecular envelope of half an amyloid fibril with caution. With this caveat in mind, we postulate that the Aβ_42_ curvilinear protofibrils, which are rich in β sheet (*7*, *32*), form an S-shaped structure similar to fibrils, with an in-register stacking of Aβ and intermolecular hydrogen bonds. The ability for the oligomers or curvilinear protofibrils to extend and elongate by templating monomer addition (Fig. 4) can be explained if, similar to fibrils, the curvilinear protofibrils have a cross-β structure. The resolution of the 3D reconstruction of the protofibrils is 1.1 nm and does not resolve cross-β structure at 0.48 nm in the power spectrum.

We postulate that the marked irregular curvature in the curvilinear protofibrils is lost when fibrils are formed. The lateral association of the protofibrils adds rigidity and strength to the structure, which also facilitates extension of the fibrils with a reduction in the tendency for fragmentation. We can infer an approximate molecular weight of the assemblies shown in Figs. 3 to 5, assuming a cross-sectional slice for each Aβ molecule of 0.48-nm thickness (which is the midpoint distance between two β strands). The smaller oligomers of Aβ (2.8 nm) are perhaps pentamers of Aβ, while a length of 4.8 nm suggests 10 Aβ molecules (45 kDa). A typical curvilinear protofibril of 48 nm in length equates to 100 Aβ molecules (450 kDa).

We also note from single-particle 2D averages that curvilinear protofibrils occasionally form tight turns that fold back on themselves to produce incomplete rings, shown in fig. S8A; this is also observed in the cryo-ET tomograms (fig. S8B). This series of incomplete ring structures suggests how annular rings, described in the next section, might also be formed from curvilinear protofibrils. According to cryo-EM single-particle analysis, the final annular structures are 5.4 nm high, twice the thickness of a curvilinear protofibril. The annular structure, described in the next section, may undergo considerable rearrangement upon inserting into the hydrophobic membrane. Conceivably, the S-shape topology could stretch out while retaining its in-register hydrogen-bonding pattern to form a β barrel structure.

### Annular structures

#### Cryo-EM

The other type of prefibrillar structure is less elongated than curvilinear protofibrils but exceeds 5 nm in all dimensions. Only two of the 10 structures generated from ab initio reconstruction of cryo-EM 2.5 million single particles fit this description—less than 20% of the lag-phase particles (fig. S7). Some of these 2D class averages (~6-nm diameter) have a ringlike appearance, which is consistent with an annular topology, previously imaged by AFM (*44*, *47*). Using this subset of 562,000 particles, three ab initio structures were generated, and one of these structures has a ringlike appearance shown in fig. S9. The 2D class averages, representing 144,000 particles, are also shown in fig. S9.

**Fig. 7.**
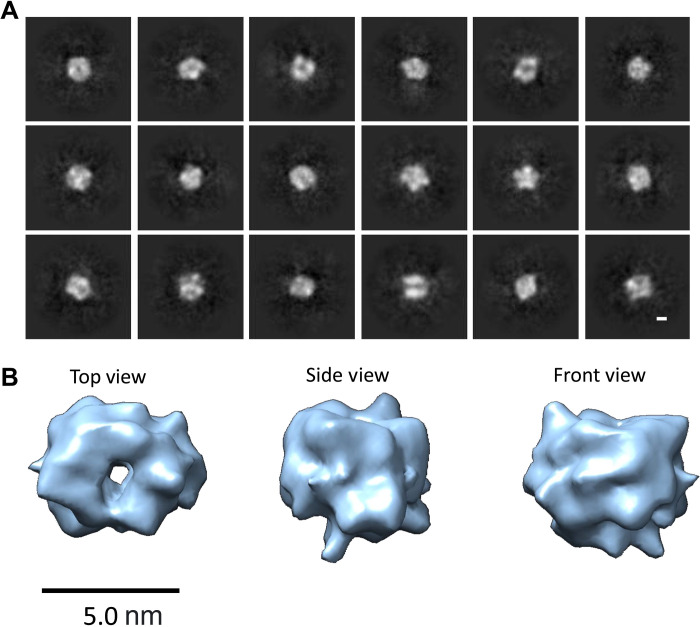
Cryo-EM 3D reconstruction of Aβ_42_ annular oligomers. 2D averages (**A**) and 3D reconstruction (**B**) from 6000 single particles suggest a structure with an internal pore. External diameter of top view is 5.5 to 6.5 nm, length of channel is 5.4 nm, and volume suggests 12 to 16 Aβ_42_ molecules. Scale bars, 5.0 nm (3D structures) and 20 Å (2D averages).

The 3D cryo-EM structure shown in fig. S9 contains an internal channel running through the middle of the structure. Inspection of the 2D class averages indicates that some of the classes do not match well with the general appearance of the 3D structure shown in fig. S9. To improve the resolution, the class averages that were poorly defined, too large, had density on the side of the ring, or suggested an incomplete ring, were manually removed, resulting in a reduced set of 22,000 particles. Inspection of the 2D class averages suggested similar structures but with more than one ring size, shown in fig. S10. The external diameter of the rings varies between 3.8 nm for the very smallest, while the majority (90%) of rings are between 5.0 to 6.5 nm. Multiple ring sizes have also been suggested from the AFM images (*44*, *47*) and reported for annular structures of the amyloid protein, α-synuclein, responsible for Parkinson’s disease (*55*). An attempt to generate two structures ab initio did not generate notably different sizes. Thus, we took this dataset and manually split it into three sets, ~7000 particles each; from these three subsets, 3D structures were generated. This pipeline is described in fig. S11. The dataset with the slightly smaller particles produced an annular structure (Fig. 7). Gold-standard Fourier shell correlation indicates a resolution of 1.1 nm, without the use of a mask. The angular distribution for these particles, which make up the 3D reconstruction, is shown in fig. S12 and indicates that there is no orientational bias in the 3D structure. Incorporation of rotational symmetry to the structure did not improve its resolution and was not applied. This 3D structure generated shows a close resemblance to the set of 2D class averages from which they are derived. We used these structures (Fig. 7) and the associated 2D class averages (fig. S14) to extract further information, in particular, the dimensions of the internal pore. The length of the pore is 5.4 nm, the external diameter of the ring is between 5.5 and 6.5 nm, and the approximate internal pore diameter is 1.0 to 1.5 nm. From the 3D structure, a volume can be derived, which will suggest a molecular weight and Aβ stoichiometry for the annular structure. The setting of the threshold (used in Fig. 7) will affect this approach, but nevertheless the structure shown has a volume of ~64,000 Å^3^, which suggests a molecular weight of 53 kDa (64,000 Å^3^ × 825 Da/Å^3^) (*56*), a dodecamer of Aβ_42_. The cryo-EM density might not include all the Aβ_42_ residues; for example, flexible N-terminal residues (residues 1 to 14) might not contribute to the structure, thus the dominant annular structure might be closer to 16 Aβ_42_ molecules. Support for the suggestion that the N-terminal residues might not contribute to the annular structures comes from images of Aβ fragment, the p3-peptide Aβ (*17*–*42*), which also produces annular assemblies of a very similar appearance (*57*, *58*).

High-resolution structures of Aβ annular oligomers, sufficient to resolve side chains, are a challenge because of their small size and structural heterogeneity. Furthermore, these structures may be quite dynamic and flexible with molten globule properties. This is suggested by multiple channel conductance, described later, which implies that the annular structures can rapidly transition in size, and this is supported by small variations in ring size from a survey of 2D class averages (figs. S10 and S13).

#### Cryo-ET

**Fig. 8.**
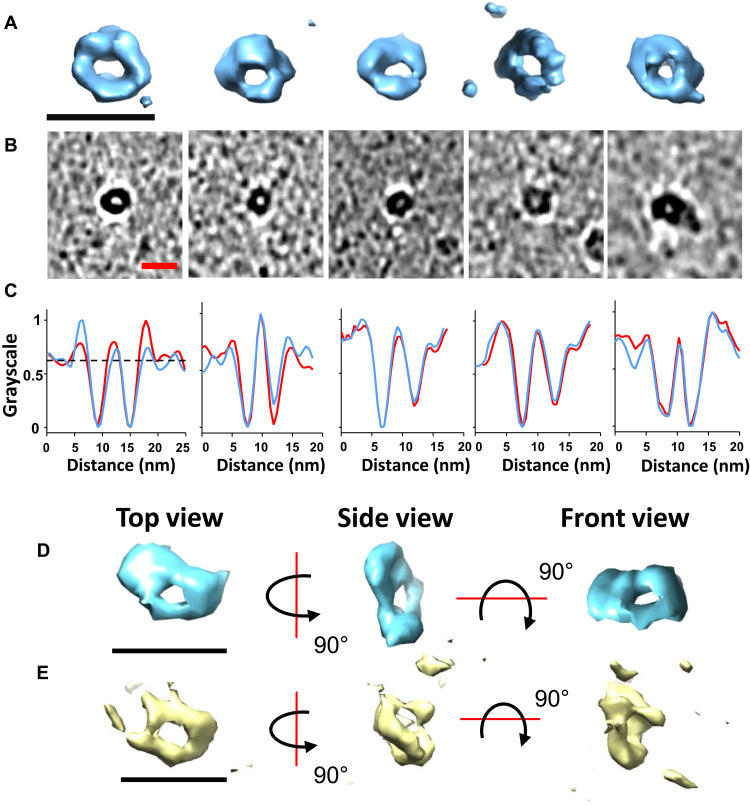
Aβ_42_ annular assemblies imaged in 3D by Cryo-ET. (**A**) Surface-rendered 3D image of five typical individual annular structures viewing down the channel. (**B**) The accompanying tomographic slice (0.54 nm thick). (**C**) Grayscale intensity profile plots, two orthogonal slices through the center of the ring. The baseline mean external diameter of the rings is 7.5 ± 0.3 nm, while the mean internal channel diameter is 2.0 ± 0.2 nm. (**D** and **E**) Top, side, and front views of two annular assemblies; external diameter of ring: ~7 nm, and internal length of channel: *~*3 nm (D) and 4 nm (E). Scale bars, 10 nm (all).

In support of the annular assemblies derived from 3D image reconstruction, we were able to identify several small ringlike structures in the cryo–electron tomograms, shown in Fig. 8. These were most abundant at a time point of assembly where curvilinear protofibrils, also present, are at a relatively early stage of extension. A surface-rendered single annular structure, imaged in 3D by cryo-ET, is shown as a supplementary movie (movie S1). The toroidal shape has an external ring diameter ranging from 7.3 to 8.1 nm (at baseline) with a mean value of 7.5 ± 0.3 nm, similar to the cryo-EM 3D reconstructions shown in Fig. 7 and fig. S9. Grayscale intensity profile plots are also shown and indicate an internal pore diameter ranging between 1.8 and 2.4 nm with a mean of 2.0 ± 0.2 nm. The length of the internal channel appears a little shorter at ~3 nm. The cryo-ET annular structures support the suggestion that these are formed by curvilinear protofibrils, which have formed a complete loop, because the height of the ring matches the 2.8-nm curvilinear protofibril diameter. Examples of curvilinear protofibrils almost forming a complete annular loop are shown in fig. S8.

Although AFM and ns-TEM imaging have previously reported structures with an annular appearance, they have remained controversial, as it has not been clear whether these structures have a channel that passes all the way through the assembly. Our cryo-ET and cryo-EM images confirm that these structures do contain a channel.

Molecular dynamics simulations have predicted stable pore structures containing 12 to 20 Aβ monomers (*59*). The dimensions of the predicted assembly with 12 Aβ molecules (*59*) match our cryo-EM structure, shown in Fig. 7. A β-hairpin barrel of Aβ has been designed using a larger heptameric scaffold protein (*60*). The template forces Aβ to form a structure with seven Aβ molecules, although there is nothing to suggest that this is an appropriate stoichiometry. The β-hairpin structures with seven Aβ molecules (PDB: 7O1Q) are compared to the annular structures determined here. Figure S14 shows that the ring of the β-hairpin structure, formed with the scaffold, is smaller than for wild-type Aβ_42_, described in Fig. 7. There has also been an NMR-based structure reported, using a designed Aβ sequence containing cysteine mutations (*35*), and the NMR restraints indicated a β barrel structure, although the NMR constraints are not sufficient to indicate the stoichiometry/size of the β barrel. An Aβ octamer, which forms an antiparallel β-sandwich structure embedded in a membrane, has also been described (*61*, *62*). Ex vivo annular assemblies have also been isolated from the AD brain and imaged by TEM, supporting a role for annular structures in AD pathology (*49*).

### Membrane permeability, ion-channel pore conductance, and the dimensions of the annular structure

**Fig. 9.**
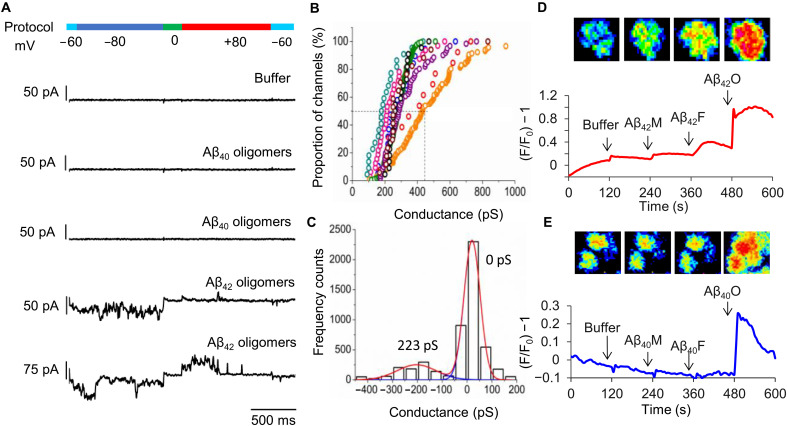
Aβ oligomer induced ion cellular influx. Patch-clamp conductance recording, voltage-clamped, for membrane excised from HEK293 cells. Only Aβ_42_ oligomers (lag phase), but not Aβ_40_, show strong conductance (**A**). Conductance from nine individual excised membranes, and each data point is the modal conductance (in picosiemens) with channel from a 1000-ms fixed voltage (**B**). Distribution of conductances over a single 1000-ms interval (**C**). Detection of intracellular Ca^2+^ in HEK293 cells by total Fluo-4 fluorescence. Also shown is single-cell fluorescence imaging of HEK293 cells after addition of buffer, monomer, fibril, and oligomer (left to right) Aβ_42_ (**D**) and Aβ_40_ (**E**). Only oligomers of both Aβ_42_ and Aβ_40_ show Ca^2+^ influx detected by Fluo-4 fluorescence.

We wanted to relate the annular structure characterized here in Figs. 7 and 8 to ion-channel membrane conductance, using patch-clamp electrophysiology. We and others have shown that Aβ can form ion-channel pores (*43*–*46*). We have shown that only Aβ_42_ oligomers, not monomers or fibrils, produce ion channels when presented to the cellular membrane (*46*). Measurements here were performed using a voltage-clamped technique with an inside-out configuration, with step voltages of −80 and +80 mV. Figure 9A shows membrane conductance induced by Aβ_42_ oligomers. This was achieved by allowing a lag-phase mixture of prefibrillar assemblies to diffuse onto the extracellular surface of the excised human embryonic kidney (HEK) 293 cellular plasma membrane. The appearance is very different from controls of buffer solution or Aβ_40_ oligomers, both of which show no membrane conductance or occasionally smaller endogenous channels with conductance of 50 to 90 pS (Fig. 9A). Aβ_42_ conductance observed is for single channels, which is indicated by conductance profiles that almost instantly become fully open or closed. The conductances produced have a particular set of properties; they are large, typically between 135 and 540 pS. They have been shown not to be ion selective (*46*) and are observed in both polarizing and depolarizing potentials. They remain open for long periods of time, often producing a “flickering” appearance. The channel conductance does not usually occur immediately upon membrane exposure to Aβ_42_ oligomers and can typically take 10 min for conductance to be observed after exposure (*46*). It appears that the Aβ_42_ annular structures, in an aqueous environment, take time to insert and perhaps undergo structural rearrangement within the hydrophobic lipid membrane to span the bilayer.

We were particularly interested in the magnitude of the conductance because this can be related to the dimensions of the channel, using the Hille equation (*63*, *64*); see Materials and Methods. The conductance values observed during the patch-clamp recordings have been ranked in size for each individual excised membrane. Conductances for nine individual excised membrane measurements are shown in Fig. 9B. Each data point in Fig. 9B is derived from a 2500-ms period of measurement, which is analyzed as conductance distribution plots, an example of which is shown in Fig. 9C. For the nine patches analyzed in detail, table S1 shows the median and upper and lower limits of conductance values. The median value for the nine channels is 320 pS. There is also considerable variation in the values for each channel; a typical range of conductance, observed between 80 and 20% of the range of values, is between 130 and 510 pS, suggesting that the channel can fluctuate in size. Some patch-clamp conductances hint at distinct pore sizes (*46*).

Assuming a channel length of 5.4 nm (Fig. 7), which is sufficient to span the lipid membrane, then a typical channel conductance of 320 pS suggests a channel diameter of 1.4 nm, which is similar to that indicated in the cryo-EM structure (Fig. 7). The range of conductance typically observed for a single channel is between 135 and 540 pS, which suggests a channel diameter of 1.0 to 2.0 nm, respectively (see table S2). The rapid changes in conductance, observed in the patch-clamp measurements, suggest that the channel is capable of rapid changes in internal diameter or the pore might be partially and transiently blocked by Aβ monomer. The similarity in the predicted and observed internal diameters implies a connection between the observed conductances and the Aβ_42_ annular structure.

We also used Fluo-4, an intracellular Ca^2+^-sensitive fluorescent dye, to measure cellular Ca^2+^ influx (Fig. 9, D and E). Unlike the Aβ ion-channel conductance, the permeability to Ca^2+^ occurs almost instantly after exposure to Aβ lag-phase oligomers. Only the lag-phase Aβ assemblies cause Ca^2+^ influx, and the addition of Aβ_42_ monomers and fibrils has minimal measurable impact on levels of Ca^2+^ influx into the cellular lumen (Fig. 9, D and E, and fig. S16). Furthermore, Ca^2+^ influx is caused by both Aβ_42_ and Aβ_40_ oligomers, while the large (135 to 540 pS) ion channels are only observed for Aβ_42_ in cellular membranes and not Aβ_40_ (Fig. 9A).

Cryo-EM and TEM images of lipid vesicles indicate that prefibrillar Aβ oligomers and curvilinear protofibrils embed and disrupt the lipid bilayer in less than 30 s after exposure to Aβ (figs. S16 and S17). The effect of the annular assemblies is very different; it typically takes 10 min for channels to form in the membrane, and the channel might remain active and open for half an hour or more. For these reasons, we suggest that this spike of Ca^2+^ influx is not the same phenomenon as the ion conductance measured by patch clamp. This implies that the instant Ca^2+^ influx is not caused by annular structures but is the result of curvilinear protofibrils inserting and carpeting the membrane, which we have previously imaged (*17*) and now show in figs. S16 and S17 with an incubation time of just 30 s.

## DISCUSSION

**Fig. 10.**
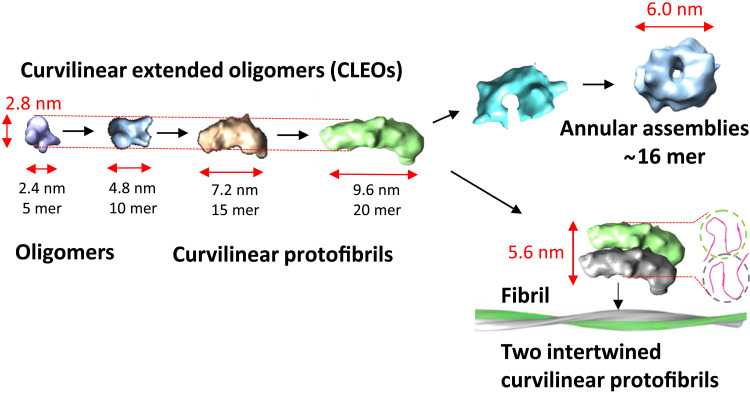
Assembly of Aβ_42_. Summary of the principle prefibrillar assemblies and their suggested conversion into fibrils. Structures are cryo-EM 3D reconstructions. 5 mer, pentamer of Aβ; 10 mer, decamer of Aβ and so on. Fibril cross section is taken from PDB: 5KK3.

Our cryo-ET, single-particle cryo-EM, AFM, and TEM imaging of the prefibrillar assemblies has enabled characterization of this group of key structures under aqueous conditions. The fibrils (*22*–*28*) and annular structures (*49*) of brain-derived ex vivo samples are similar to in vitro Aβ structures reported. This suggests that Aβ assembly under in vitro aqueous conditions can parallel in vivo Aβ assembly. The collection of structures observed suggests a mechanism of assembly, which points to an extension from small oligomers to curvilinear protofibrils, with a consistent 2.8-nm diameter. The specific bidirectional templated addition of Aβ onto the end of these small oligomers has been shown directly in real time by AFM. The distinction between small oligomers and curvilinear protofibrils, both 2.8 nm in diameter, is not particularly meaningful, as these structures are a continuum. We suggest that the curvilinear protofibrils should be referred to as extended oligomers. These make up 80% of the prefibrillar assemblies found in the micrographs. Both oligomers and curvilinear extended oligomers (CLEOs) exhibit the same type of interaction with lipid membranes, both inserting into the upper leaflet of the bilayer to the same extent, carpeting the surface causing permeability across the membrane (*17*–*19*, *65*). When viewed in the round, cytotoxicity of both oligomers and CLEOs suggests a similar membrane interaction, permeability, and cytotoxicity (*14*, *18*). Furthermore, the structural dimensions of the CLEOs hint at a mechanism by which fibrils can form. In particular, lateral association of two CLEOs forms a structure similar in dimensions to that of a basic amyloid fibril, summarized in Fig. 10.

Furthermore, our cryo-ET 3D tomograms and single-particle cryo-EM 3D reconstruction indicate that annular structures do form a channel that can potentially span lipid bilayers. The magnitude of the ion-channel pore conductance is a strong link to the dimensions of the annular structure. In the future, we hope that it may be possible to obtain atomic resolution structures, embedded in lipid bilayers. In general, annular structures are formed for at least seven different amyloid-forming proteins (*47*, *57*). Annular structures, believed to be responsible for Parkinson’s disease, have been reported for α-synuclein and imaged by cryo-EM at a resolution insufficient to resolve secondary structure (*55*). Similar to Aβ, α-synuclein has also been shown to form ion-channel pores (*66*, *67*). Molecules, which block the annular channels or inhibit their formation, are potential therapeutics for treating AD (*45*, *68*, *69*).

## MATERIALS AND METHODS

### Aβ sample preparation

Aβ wild-type peptides (Aβ_42_ and Aβ_40_) were purchased from GenScript and EZBiolab, which were synthesized by *N*-(9-fluorenyl)methoxycarbonyl chemistry and purified with reverse-phase high-performance liquid chromatography, and the sequences were verified by electrospray ionization mass spectrometry. The lyophilized Aβ peptides were solubilized at 0.7 mg ml^−1^ (or 2 mg ml^−1^ for cryo-EM) in ultrapure water at pH 10. Aβ monomers were isolated by size exclusion chromatography (SEC) using a Superdex 75 10/300 GL column. Concentrations of Aβ stock solutions were determined by absorbance using a spectrophotometer at 280 nm (ε = 1280 M^−1^ cm^−1^).

### Fibril growth assay

Aβ stock solution was diluted into 10 μM by buffer (160 mM NaCl and 50 mM Hepes at pH 7.4), aliquoted into a 96-well plate, and then placed into a fluorescent well-plate reader (FLUOstar Omega, BMG Labtech, Aylesbury, UK). Well plates were set at a fluorescence reading every 30 min, fluorescence excitation at 440 nm, and emission at 490 nm. Aβ fibril formation was monitored by ThT fluorescent dye in separate wells and under quiescent conditions. Aβ oligomers were obtained from the well plate at the end of the lag phase (~10% max ThT signal), with ThT in adjacent wells. The lag-phase oligomer preparations were immediately used or stored at −80°C for use.

### Cryo-ET

Lag-phase oligomers of Aβ_42_ (5 μM, monomer equivalent) in Hepes buffer (30 mM) and NaCl (160 mM) were applied to QUANTIFOIL R2/2 holey carbon grids and then plunge frozen using a Thermo Fisher Scientific Vitrobot. Cryo-ET images were obtained with a Thermo Fisher Scientific Glacios TEM operating at 200 kV, using a 4000 × 4000 Falcon 3EC direct electron detection camera, with a magnification of 73,000. Pixel size was 1.9 Å at the specimen level. Tilt series were obtained between −60° and +60° with increments of 3° using the dose symmetric scheme. The defocus was set between 3 and 4 μm, and the total dose was ~100 e/Å^2^. Typically, tomographic images shown are a sum of 10 slices (7.6 nm thick). 3D tomograms were reconstructed from the tilt series using RAPTOR (*70*) and the IMOD software package, followed by weighted back projection or simultaneous iterative reconstruction technique, with the TOMO3D package (*71*, *72*). Final tomograms were binned 4×, resulting in a pixel size of 7.6 Å. Images of a single threshold surface were produced using Chimera (*73*) following a 3 × 3 × 3 median filtering operation. The lengths of curvilinear protofibrils, in three dimensions, were obtained by manual inspection in Fiji (*74*) of volume regions previously rotated and extracted from full tomograms in 3dmod (slicer module).

### Cryo-EM

#### *A*β*_42_ oligomer preparation for cryo-EM*

Lyophilized peptides were solubilized at 2 mg ml^−1^ in ultrapure water at pH 10. Aβ_42_ monomer (300 μM) was incubated with NaCl (160 mM) and Hepes (50 mM) buffer at pH 7.4 at room temperature, after vortexing for 15 s. Aβ_42_ prefibrillar assemblies with predominantly oligomeric and curvilinear protofibril structures were obtained after 15-min incubation, confirmed by negatively stained EM. “Lag-phase” oligomeric samples were ready to be loaded onto cryo-grids immediately.

#### Cryo-EM

Holey carbon grids (QUANTIFOIL Au R 2/2, 200 mesh) were glow discharged with easiGlow at 20 mA for 45 s. Aliquots (4 μl) of Aβ_42_ oligomers were applied to the grids and blotted for 2.6 s with filter paper (Whatman) at 75% humidity and 4°C using a Leica EM GP2 plunge freezer. Sample grids were prescreened on JEOL JEM-2100Plus. The dataset was acquired on a Thermo Fisher Scientific Titan Krios microscope, 300 kV, with Gatan K3 detectors. Data were acquired with an aberration-free image shift, using EPU software. In total, 13,843 movies were collected, at a superresolution of 0.55 Å/pixel, with a total dose of 40 e/Å^2^ and defocus range between 0.5 and 3.5 μm.

#### Data processing

All superresolution frames were gain corrected, binned, aligned, and dose weighted; this was followed by summation into a single micrograph using motion correction in RELION (*75*). The pixel size was binned to 1.1 Å/pixel. The estimation of parameters for the contrast transfer function (CTF) was carried out using CTFFIND4.1 in RELION. Initially, manually selected particles from 50 micrographs were used to generate a model for automatic particle picking using crYOLO. Further processing was performed in CryoSPARC (*76*). Particles were binned and extracted at 2.2 Å/pixel. 2D classification was performed from 3 million extracted particles. A workflow is shown in fig. S11. 3D models were reconstructed de novo from 2D class averages. Nonuniform refinement was used to improve the resolution. Molecular graphics and analyses were performed in Chimera (UCSF) (*77*). A protofibril model was fitted into an S-shaped half map of a mature fibril (PDB: 2MXU) (*23*).

### Atomic force microscopy

Aβ_42_ samples were collected during the early lag phase, before any detectable ThT signal. The samples were deposited onto mica at an initial concentration of 15 μM and immediately diluted to 5 μM, yielding a total volume of 3 ml in phosphate-buffered saline (PBS) buffer [137 mM NaCl, 2.7 mM KCl, and 10 mM phosphate buffer (pH 7.4)]. Mica discs, sourced from Agar Scientific Ltd., were cleaved five times using sticky tape and affixed to a surface with ultraviolet structural adhesive (Loctite AA 358).

AFM experiments were conducted using a NanoWizard 4 BioScience AFM (JPK Instruments, Germany) integrated into an iX81 optical microscope frame (Olympus, Belgium). High-resolution topographical imaging of Aβ oligomers and fibrils was performed using a silicon nitride tip mounted on a soft, gold-coated triangular silicon nitride cantilever (MLCT-BIO, cantilever C, Bruker). The tip had a nominal radius of 20 nm, and the cantilever exhibited a nominal spring constant of 0.01 N/m. Samples on mica were imaged in aqueous solution at a controlled room temperature of 22°C.

AFM imaging was conducted in contact mode using JPK software, with a resolution of 512 or 1024 samples per line and a scan rate of 1 Hz. This setup enabled the acquisition of 10 μm by 10 μm micrographs every 8 min, with a pixel resolution of 9 to 19 nm. Image analysis was carried out using JPKSPM Data Processing software, applying first-order flattening and plane fitting. The height scale was adjusted to enhance visualization of Aβ prefibrillar structures, typically displaying a height range of 0.0 to 4.0 nm.

### Time course of assembly linked with ThT signal and TEM

To image Aβ assembly over time using TEM Aβ_42_ fibril assemblies, samples were generated in a well plate alongside Aβ_42_ fibril growth assay but without addition of ThT dye. SEC-purified Aβ_42_ monomer (10 μM, 300 μl) was added to the 96-well plate, and 5-μl Aβ_42_ aliquot samples were collected at different time points, which were 0 min, 28 hours (end of lag-phase time point), 30 hours, 32 hours, 34 hours, 36 hours, 38 hours, 50 hours (midpoint of maximum ThT signal), and 84 hours (equilibrium phase).

Carbon-coated copper grids, 300 mesh, (Agar Scientific Ltd.) were glow discharged by the easiGlow glow discharge system (PELCO Inc., USA). Aβ_42_ samples (5-μl aliquot) were added onto the glow discharged grids using the droplet method and blotted after 1 min. Four microliters of uranyl acetate [1% (w/v)] was used to negatively stain the samples, then blotted, and rinsed after 30 s at room temperature. A JEOL JEM-1400Flash electron microscope (JEOL Ltd., Japan) at 20,000 magnification was operated at 120 kV, paired with a JEOL Matataki camera and corresponding microscope image analysis software (JEOL).

For analyzing Aβ_42_ assemblies’ lengths, four micrographs for each time point were divided into four equal quadrants, and the lengths of all the particles were measured from one of the quadrants by Fiji ImageJ software. Typically, ~100 particles were measured for each time point.

### Patch-clamp conductance measurements

#### Cell culture

HEK293 immortal cells were incubated at 37°C in a 5% CO_2_ incubator in Dulbecco’s modified Eagle’s medium (DMEM) supplemented with 10% fetal bovine serum (200 U) and penicillin-streptomycin (0.2 mg/ml) in a 30-ml cell culture flask. Cells were split at ~70 to 80% confluency around every 5 to 7 days using Ca^2+^- and Mg^2+^-free PBS (pH 7.2), during which a fraction of cells were plated in 35-mm diameter Easy-Grip petri dishes with the same buffer and incubated for 2 to 3 days before use. Reagents and media were purchased from Sigma-Aldrich and Thermo Fisher Scientific (Invitrogen).

#### Patch-clamp recordings

All patch-clamp measurements were made from excised membrane in an inside-out configuration. This allows Aβ to diffuse to the extracellular surface of the HEK293 cell plasma membrane, in voltage-clamp mode. Tip-burnt, polished microglass pipettes (GC150TF-10, Harvard Apparatus) were backfilled with extracellular buffer (containing 121.4 mM NaCl, 10 mM CsCl, 9 mM Na-Hepes, 1.85 mM CaCl_2_, 1.87 mM MgCl_2_, 2.16 mM KCl, and 0.1 mM EGTA) with the addition of Aβ oligomers of 5 μM (monomer equivalent) at pH 7.4. Aβ oligomers were able to diffuse toward the extracellular membrane surface within the pipette. The excised membrane patches were ~1 to 3 μm in diameter. The pipette resistance varied from 2 to 6 megohm when filled with recording solution. Junction potentials appeared at boundaries of ionic asymmetry and were considered for using an applied pipette offset potential. Recordings were sampled at a rate of 2 kHz with 500-μs intervals with a low-pass 8-pole Bessel filter frequency of 0.2 kHz. The holding potential was set to −60 mV.

Channel-induced transmembrane currents were recorded by voltage clamping for 30 to 45 min. A protocol from −80 to 0 to +80 mV using an Axopatch 200B amplifier (Axon Instruments) was applied using pCLAMP 11 software. All recordings were processed using Clampfit software, and a low-pass boxcar filter was applied.

#### Determining conductance values

Current/voltage was used to calculate channel conductance (in picosiemens). Distribution plots of conductance for a 2500-ms interval were plotted; see Fig. 9C. These were used to obtain typical (modal) conductance values of 2500-ms intervals and used as a single data point for the total recording. Typical 40 to 100 data points for each excised membrane were obtained (Fig. 9B).

#### Calculation of channel pore diameter using channel conductance

To calculate the theoretical pore diameter, a model developed by Hille (*63*) and adapted by Cruickshank (*64*) was adopted. Pore diameter (*d*) was calculated by ionic conductance (*g*), pore length (*l*), and solution resistivity (ρ) of 80 ohm·cm (*78*) (Eq. 1)

$$d=\frac{\rho g}{\pi }\left(\frac{\pi }{2}+\sqrt{\frac{\pi^{2}}{4}+\frac{4\pi l}{\rho g}}\right)$$ (1)

### Cellular Ca^2+^ influx

#### Fluo-4 AM–loaded HEK293 cells

HEK293 cells were maintained at 37°C in 5% CO_2_ containing DMEM (purchased from Thermo Fisher Scientific) supplemented with 10% fetal bovine serum and penicillin-streptomycin (0.2 mg/ml). Cells were plated into a 12-well plate, 1 ml in each well, and incubated overnight; cells typically gained ~50% confluence. Next, DMEM was replaced with fresh DMEM, which was supplemented with 5 μM Fluo-4 AM (Abcam). Plates were then left in an incubator for a further 40 min to enable the cellular uptake of the Ca^2+^-sensitive fluorescent dye. Excess extracellular Fluo-4 AM was removed by two washes of 400 μl of DMEM Eagle’s cell medium in each well. Cells were then incubated for a further 20 min at 37°C to allow deesterification of intracellular Fluo-4 to occur, which activates Ca^2+^-dependent fluorescence. Last, the DMEM was replaced with an aqueous buffer containing 121 mM NaCl, 10 mM CsCl, 9 mM Hepes, 1.85 mM CaCl_2_, 1.87 mM MgCl_2_, and 2.16 mM KCl, buffered to pH 7.4, to mimic conditions used for patch-clamp recordings. The Fluo-4–loaded cells were then ready for time-lapse fluorescence microscopy imaging.

#### Ca^2+^ fluorescence imaging of Fluo-4 AM

Fluorescence microscopy was performed using an inverted Leica DM IL microscope with ×10 or ×20 magnification. The band-pass filter allowed excitation at 470 nm, and emission was recorded at 520 nm. Time-lapse fluorescence images and bright-field visible light images were acquired using a charge-coupled device camera with a temporal resolution of one image every 5 s; recordings were for 10 to 30 min.

Three Aβ_42_ and Aβ_40_ preparations were studied: monomers (SEC purified), oligomers (from the end of the lag phase), and fibrils (from the plateau phase). Stock solutions of 30 μM Aβ were added to HEK293 cells within 450 μl of buffer to produce a final Aβ concentration of 5 μM. The microscope was operated using ProgRes CapturePro 2.8.8 software, and fluorescence intensity was measured via ImageJ. Fluorescence intensities were measured by analyzing the overall field, using the time series analyzer V3 plugin. Individual single-cell fluorescence intensities reported similar Ca^2+^ influx behavior. Changes in Fluo-4 fluorescence signals with time were presented as (*F*/*F*_0_) – 1, where (*F*) is the observed fluorescence and (*F*_0_) is the background fluorescence at a time point just before the addition of Aβ. Typically, each condition was recorded using four separate wells, with at least three independent repeats. Ionomycin (Merck Millipore) disrupts cellular membrane integrity and was used as a positive control to confirm consistent cellular incorporation of Fluo-4 AM.

### Lipid vesicle preparation and EM imaging

Large unilamellar vesicles (LUVs) were produced using an extrusion method. The lipids used a mixture of 68:30:2 by weight of phosphatidylcholine:cholesterol:monosialotetrahexosyl-ganglioside; for further details, see (*17*). After air drying, the lipid film was resolubilized in aqueous buffer, NaCl (160 mM), and Hepes (30 mM) buffered at pH 7.4, to a lipid concentration of 1 mg ml^−1^. LUVs were formed from solubilized lipids using a benchtop mini extruder (Avanti Polar Lipids, Alabama, USA). The lipid solution was passed across a polycarbonate membrane (Cytiva Whatman, USA), with a 100-nm pore size, 21 times at a temperature of 65°C.

Cryo-EM imaging of the vesicles in the absence and presence of Aβ_42_ oligomers (20 μM monomer equivalent, obtained at the end of the lag phase) was incubated with the lipid vesicles (0.5 mg ml^−1^) for just 30 s. The Lacey carbon grids (300 mesh) (QUANTIFOIL) were glow discharged with PELCO easiGlow at 20 mA for 45 s. A 4-μl aliquot of the vesicles was applied to the grids and blotted for 2.4 s with filter paper (Whatman; level 1) at 75% humidity and 4°C using a Leica EM GP2 plunge freezer. JEM-2100Plus (JEOL Ltd., Japan), operated at 200 kV, fitted with a Gatan OneView camera, was used to image the vesicles.

Vesicles (0.05 mg ml^−1^) were also imaged by TEM, negatively stained in the absence and presence of Aβ_42_ oligomers (10 μM monomer equivalent, obtained at the end of the lag phase), and incubated with the lipid vesicles for just 30 s. Then 5-μl aliquots of sample were added to glow discharged Lacey carbon–coated 300-mesh grids (Agar Scientific Ltd.) by the droplet method, then blotted after 30 s, and rinsed with ddH_2_0. Following this, 4 μl of uranyl acetate (1 g/100 ml) was added, then blotted after 15 s, and rinsed with ddH_2_O. Images were taken from a JEM-2100Plus electron microscope (JEOL Ltd., Japan), operated at 200 kV, and fitted with a Gatan OneView camera at ×50,000 magnification.
