# Supplementary material for: Amyloid-β oligomers, curvilinear and annular assemblies, imaged by cryo-ET, cryo-EM, and AFM
Source: Sci Adv. 2025 Aug 27;11(35):eadx9030. doi: 10.1126/sciadv.adx9030 (PMC12383235; doi:10.1126/sciadv.adx9030)
Supplement: Supplementary file 1 — Figs. S1 to S17 Tables S1 and S2 Legend for movie S1 [file sciadv.adx9030_sm.pdf]

Science Advances
AAAS

Supplementary Materials for

Amyloid-β oligomers, curvilinear and annular assemblies, imaged by cryo-ET, cryo-EM, and AFM

Ruina Liang *et al.*

Corresponding author: Vidya C. Darbari, v.darbari@qmul.ac.uk; John H. Viles, j.viles@qmul.ac.uk

Sci. Adv. **11**, eadx9030 (2025)
DOI: 10.1126/sciadv.adx9030

The PDF file includes:

Figs. S1 to S17
Tables S1 and S2
Legend for movie S1

Other Supplementary Material for this manuscript includes the following:

Movie S1

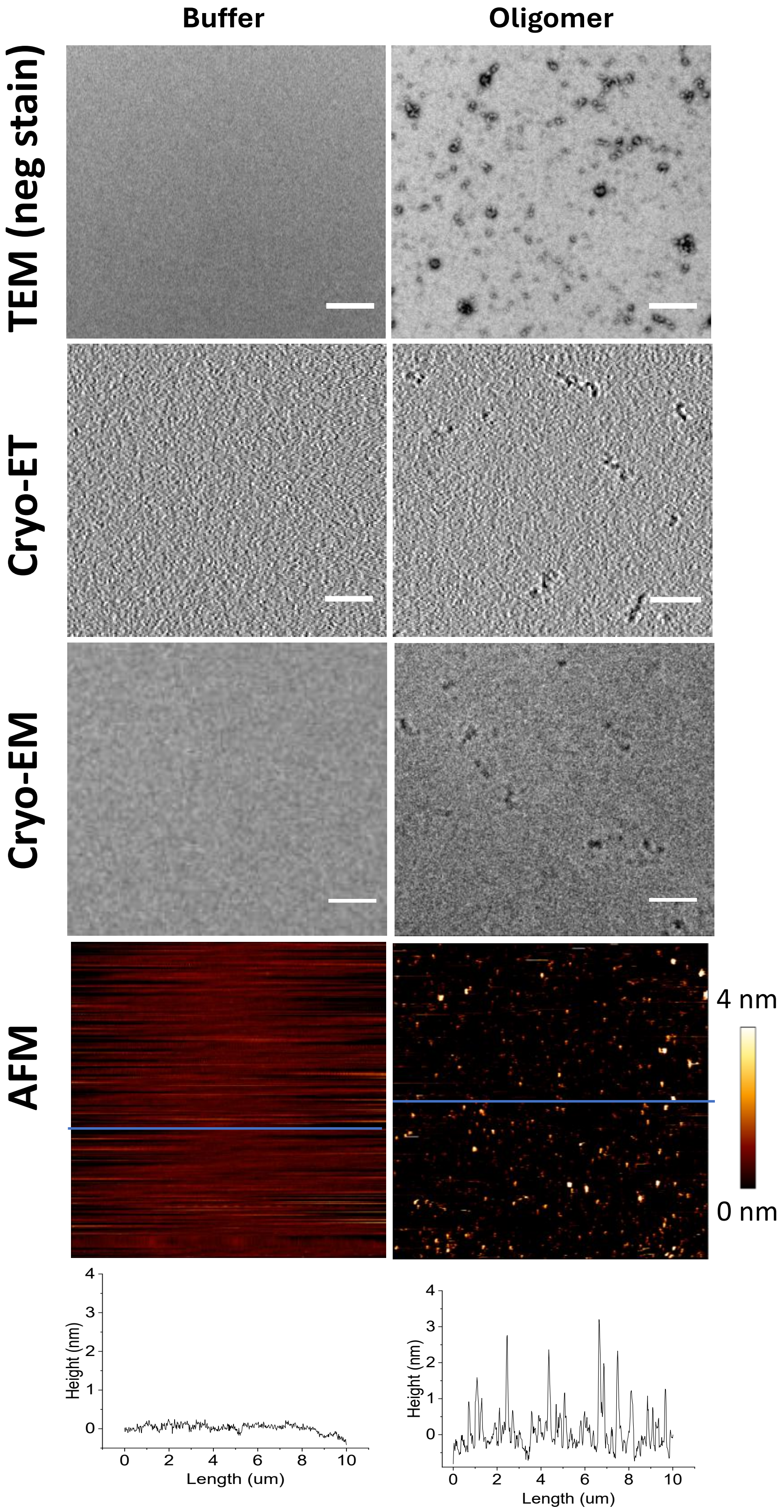

Supplemental Figure S1. Comparison of buffer-only and Aβ_{42} oligomers. Micrographs are obtained using four imaging techniques. Appearance of a micrograph with just buffer present (left column). Oligomers were taken towards the end of the lag-phase of Aβ_{42} assembly (right side). Scale bars TEM 200 nm; cryo-EM 20 nm; cryo-ET 50 nm; AFM images are 10x10 µm. Early-stage oligomers are only 3 nm in diameter but are distinguishable from contaminates in buffer-only preparations. Aβ_{42} oligomer sample is 10 µM (monomer equivalent) form TEM with uranyl acetate negatively-stained; Cryo-ET (Aβ_{42} 5 µM); Cryo-EM, (Aβ_{42} 75 µM), AFM, (Aβ_{42} 5 µM) . Buffer is pH 7.4 PBS

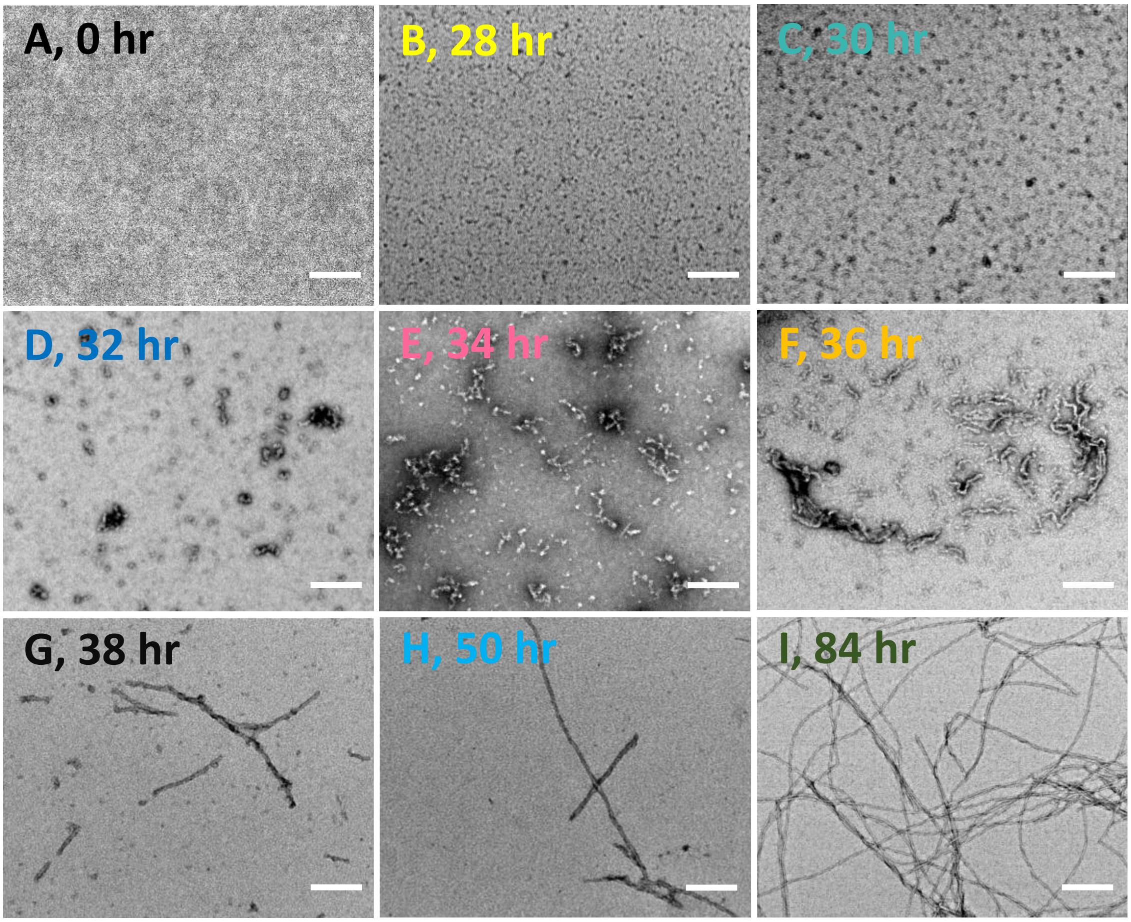

Supplemental Fig S2. TEM images of Aβ_{42} assemblies at 9 time points. (A) monomer at time = 0 hrs, (B) t= 28h, (C) t= 30h, (D) t= 32h, (E) t= 34h, (F) t= 36h, (G) t= 38h, (H) t= 50h and (I) t= 84h. Fibrils are negatively stained with 1% uranyl acetate, incubated quiescently from 10 µM SEC monomer peptide, pH 7.4. Scale bar = 200 nm, Mag 20K. (J) Kinetic trace of SEC purified Aβ_{42} (10 µM) incubated with ThT (20 µm), HEPES buffer (50 mM, pH 7.4) at 30°C.

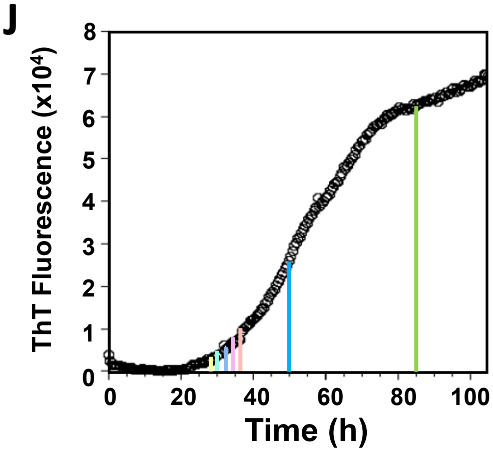

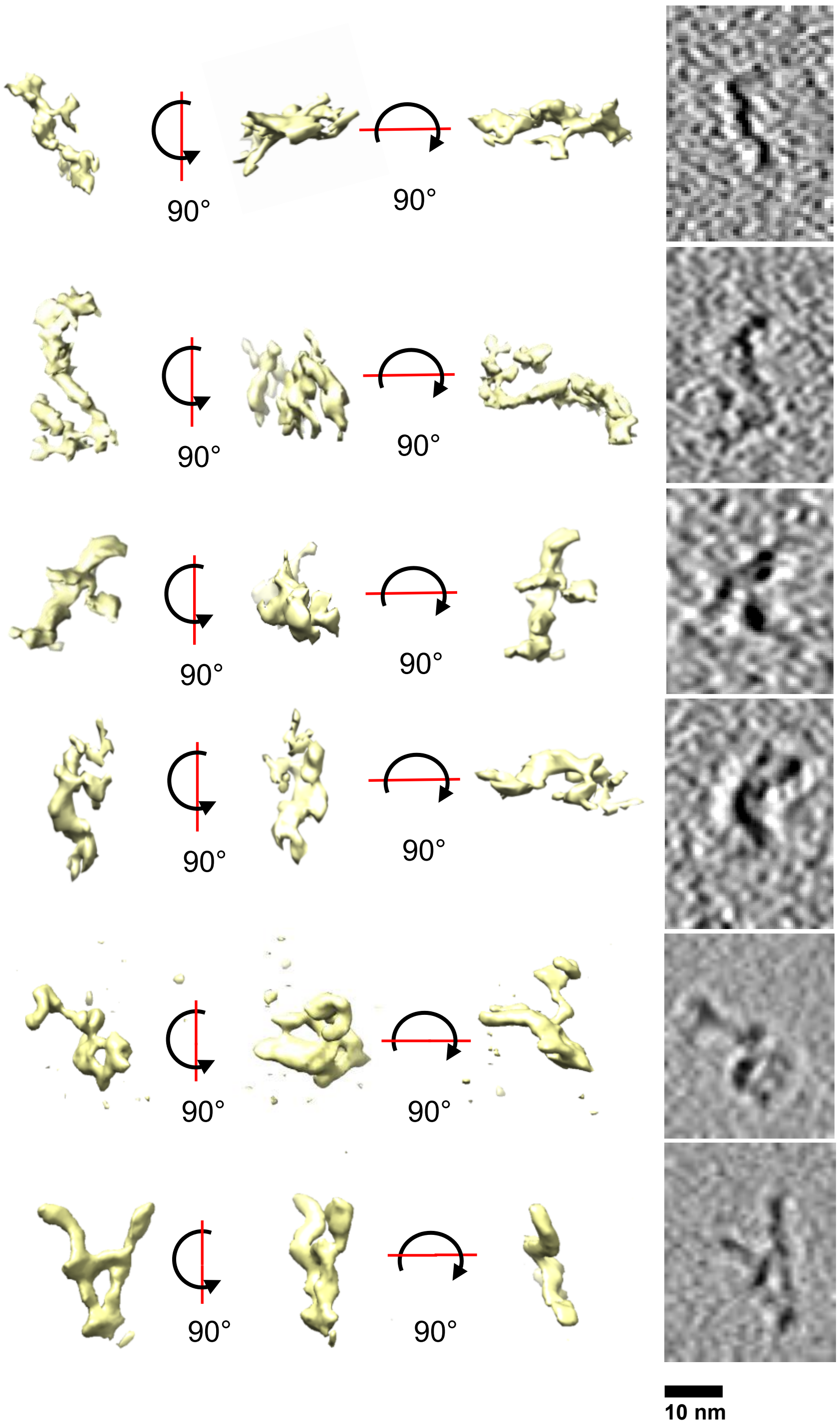

Supplemental Figure S3: Cryo-ET images, examples of irregular curvy and branched appearance of curvilinear protofibrils. Diameter of assemblies are *ca.* 2.8 nm. Tomogram slices 7.8 Å thick, scale bar 10 nm.

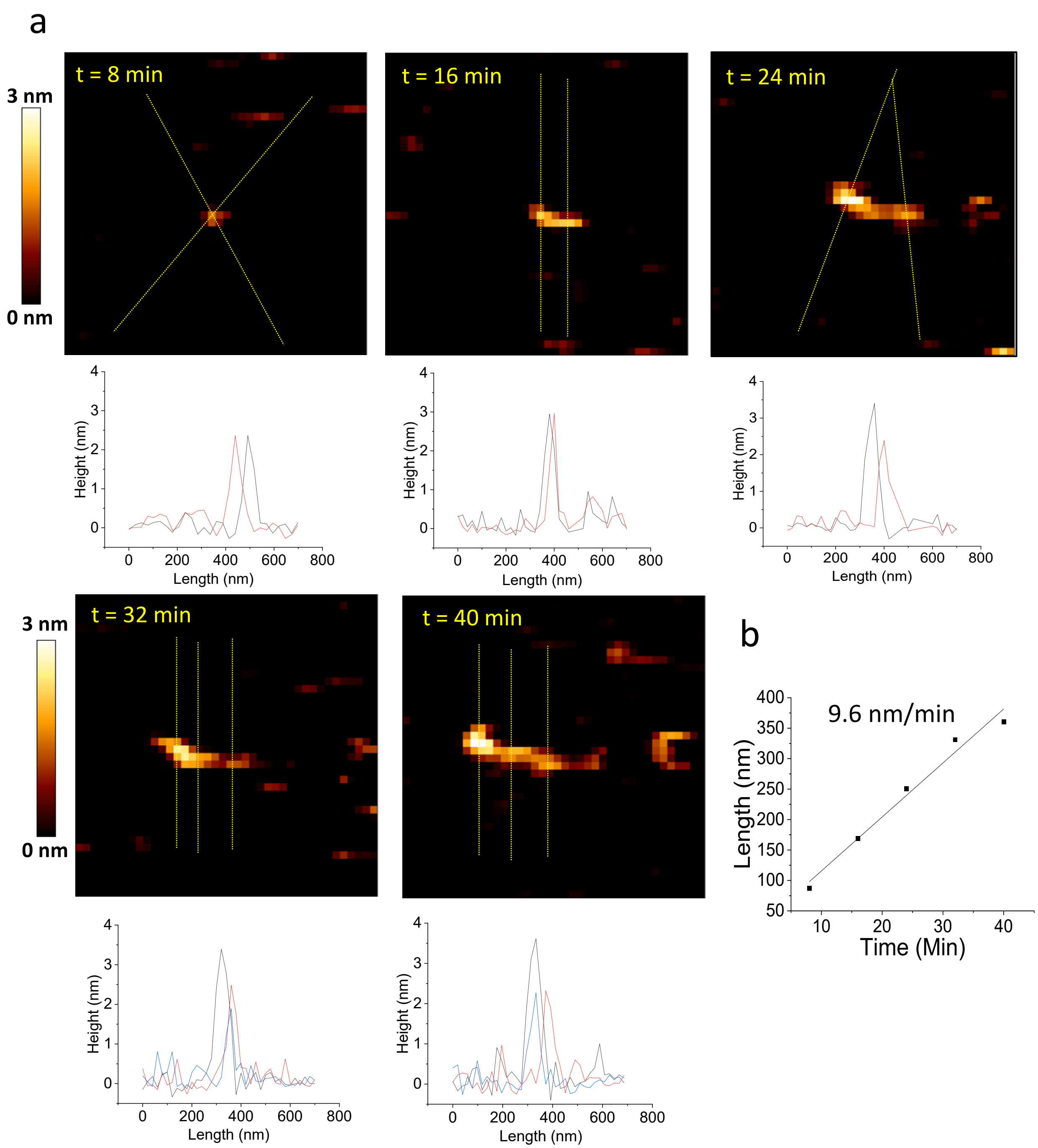

Supplemental Figure S4: Real time extension of oligomer to form curvilinear protofibrils. a) AFM images on mica support in Aβ_{42} solution (5 μM). Images recorded every 8 mins. Height of oligomers and extending curvilinear protofibrils remains at typically close to 2.8 nm. Cross-sectional height shown for each image as dashed line. Each micrograph is 783x783 nm, with a height of 0.0-3.0 nm. Aβ_{42} (5 μM) growth on mica support at 22 ^{o}C, pH 7.4, PBS. b) The length of the extending curvilinear oligomers has been plotted *versus* time. Linear best-fit line indicate a rate of oligomer extension of 9.6 nm min^{-1}.

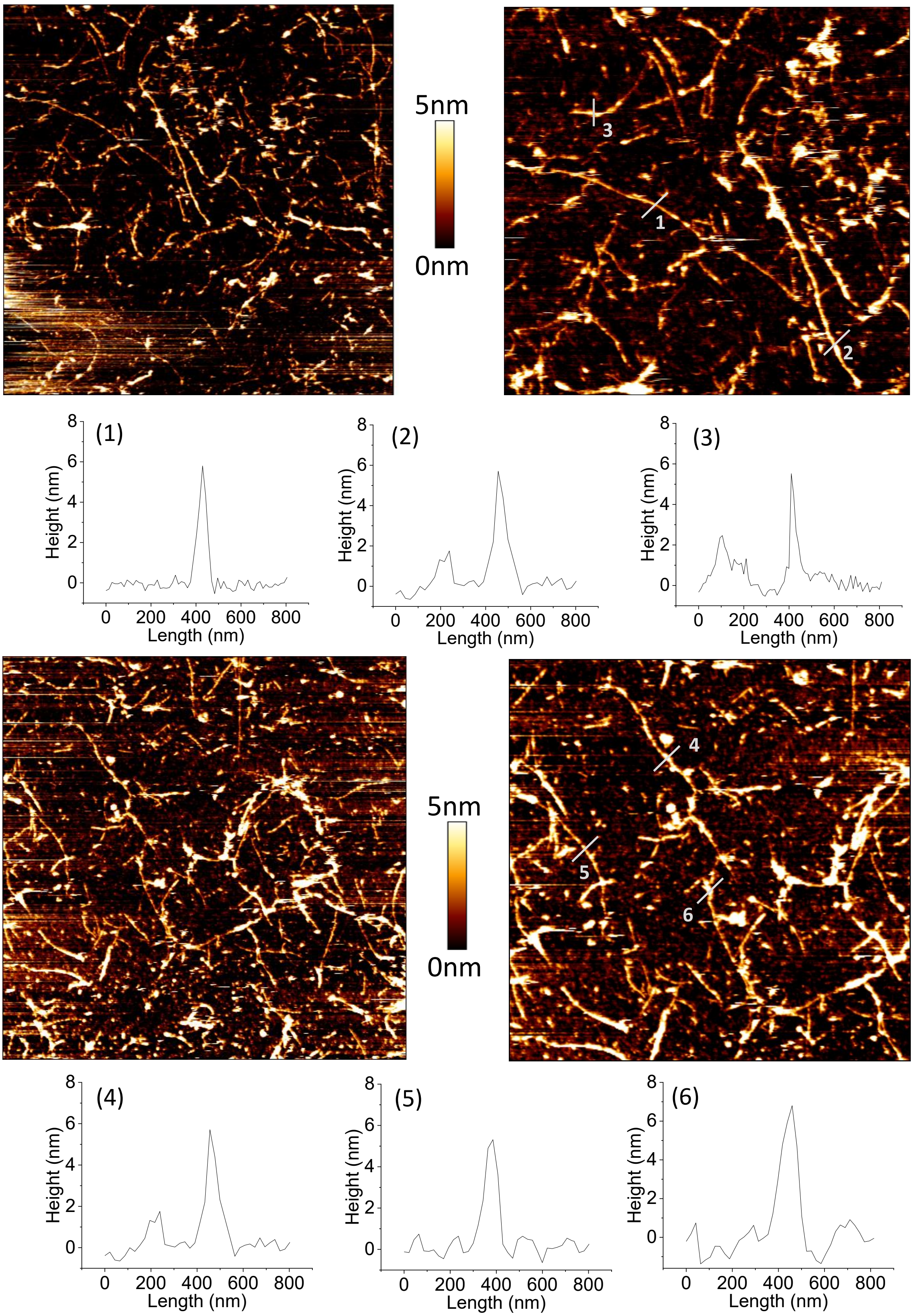

Supplemental Figure S5: AFM images of Aβ_{42} fibrils. Aβ_{42} assemblies taken at plateau stage of fibril formation (5 μM), 72 hr incubation. Relatively long and straight fibril structures, consistently 6 nm in height. Height profiles of six typical fibrils are shown. Micrographs on left are 10 by 10 μm. Expansion are on right, 5.86 by 5.86 μm. Imaged on mica support in Aβ_{42} PBS solution, pH 7.4.

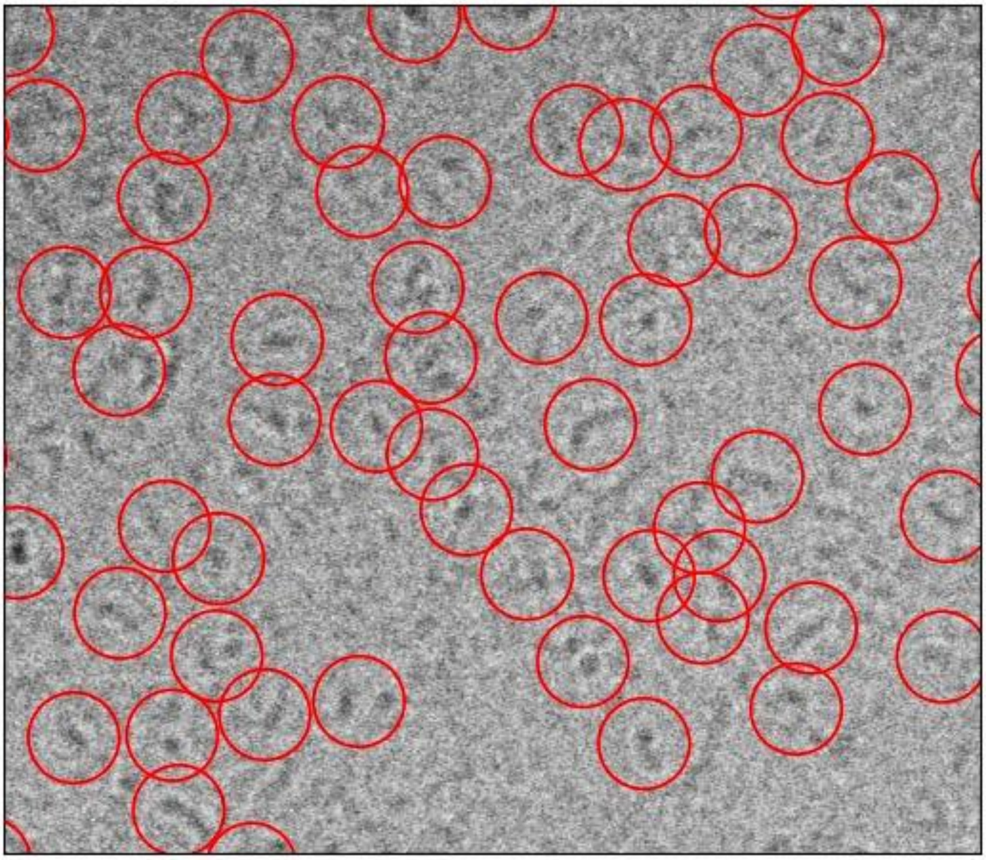

Supplemental Figure S6: **Typical cryo-EM micrograph**. Circles are set at 10 nm, highlighting the range of oligomers and curvilinear protofibrils. Three million particles were auto-picked, all the various forms of Aβ particles were picked simultaneously using Cryolo. (Template auto-picking was not used). Lag-phase Aβ42 assemblies 300 μM incubated for 30 mins.

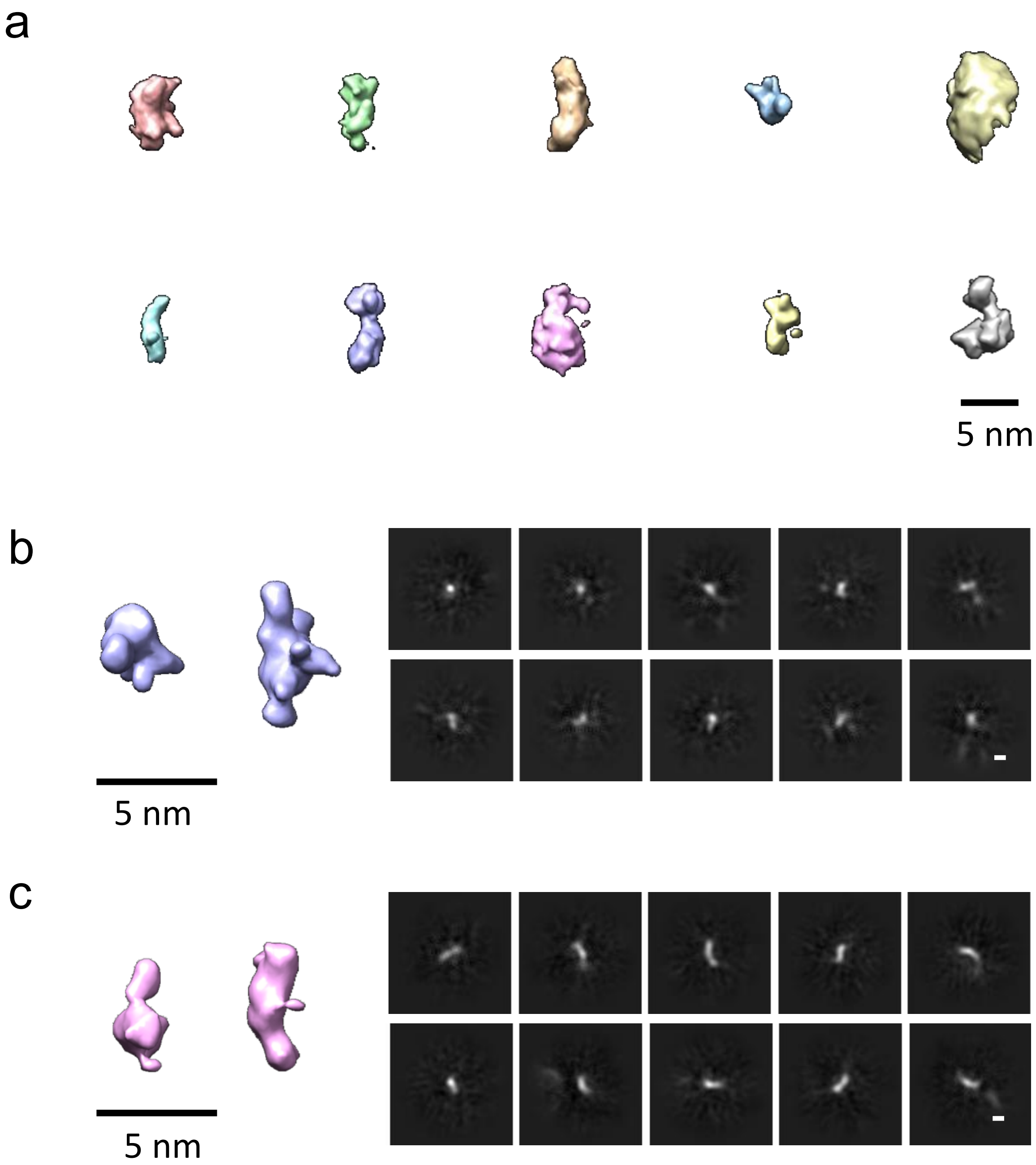

Supplemental Figure S7a-c: 3D *ab-initio* reconstruction for Aβ_{42} oligomers, prefibrillar assemblies. a) Ten unique 3D structures generated from 2.5 million particles; between 176k and 499k particles for each structure b) & c) two individual 3D *ab-initio* models representing the smaller oligomer/protofibrils, together with representative 2D averages. Scale bar: 5 nm for 3D structures, 2 nm for 2D averages.

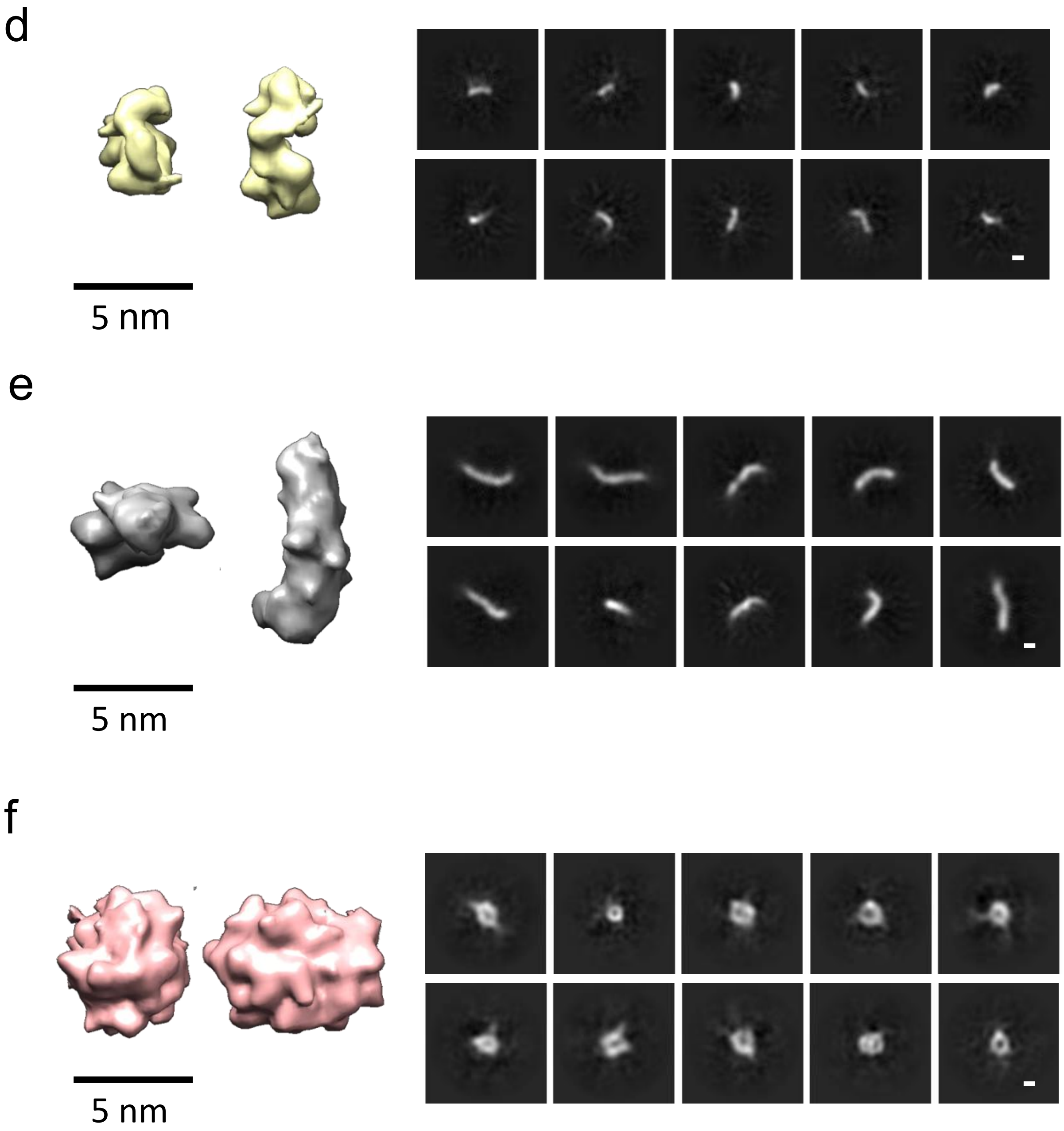

Supplemental Figure S7d-f: 3D *ab-initio* reconstruction for Aβ42 oligomers, prefibrillar assemblies. Showing three of the ten structures generated. d) & e) protofibrils, together with representative 2D averages. f) larger assemblies with all dimensions greater than 5 nm, for this structure, some of the 2D averages have a ring like appearance. Scale bar: 5 nm for 3D structures, 2 nm for 2D averages.

a

b

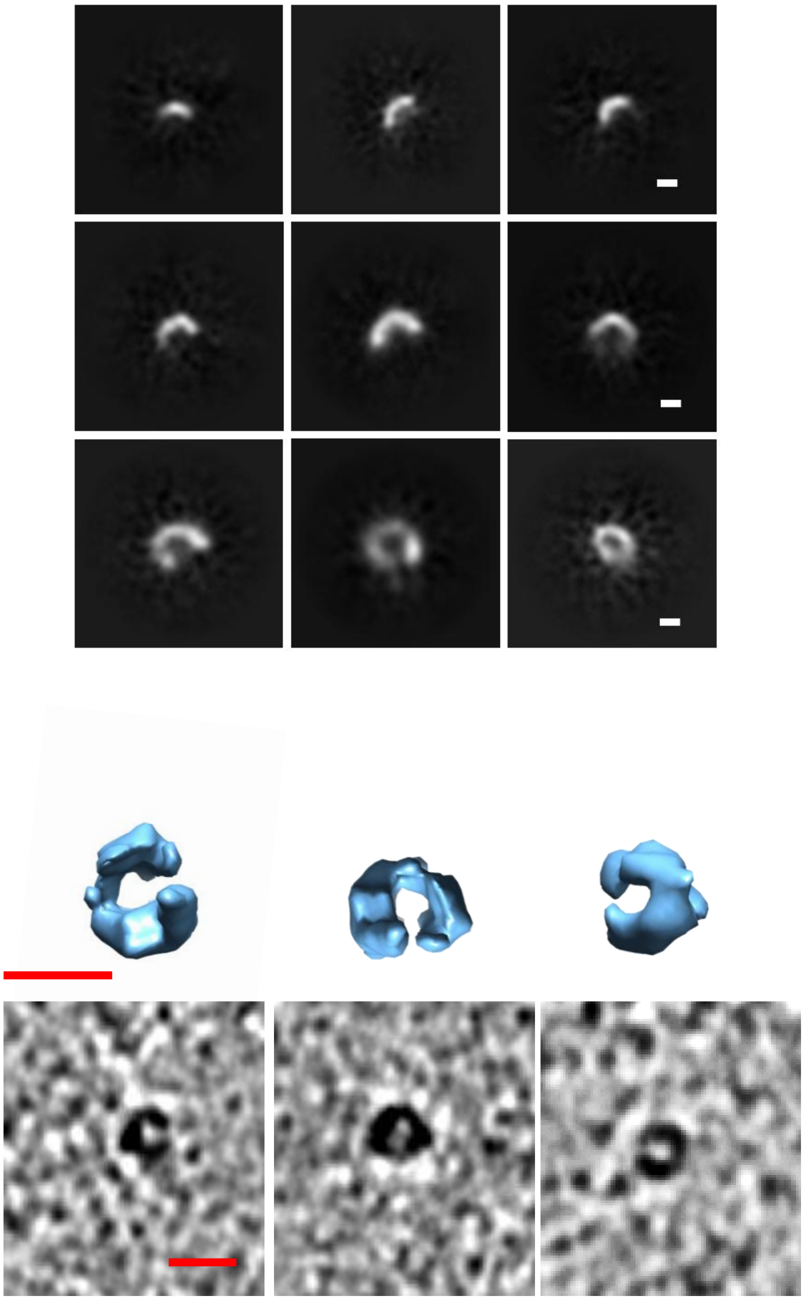

Supplemental Figure S8 . Incomplete curvilinear protofibrils loops suggest mechanism of annular formation. (a) Selected curvilinear protofibrils with a marked curvatures and ordered by length. Cryo-EM 2D class averages of *ca*. 21,000 particles per image, scale bar 2 nm. (b) Examples of curvilinear protofibrils with a marked curvatures, from cryo-ET. Surface rendered 3D assembles with accompanying tomogram slice 0.54 nm thick, scale bar 10 nm.

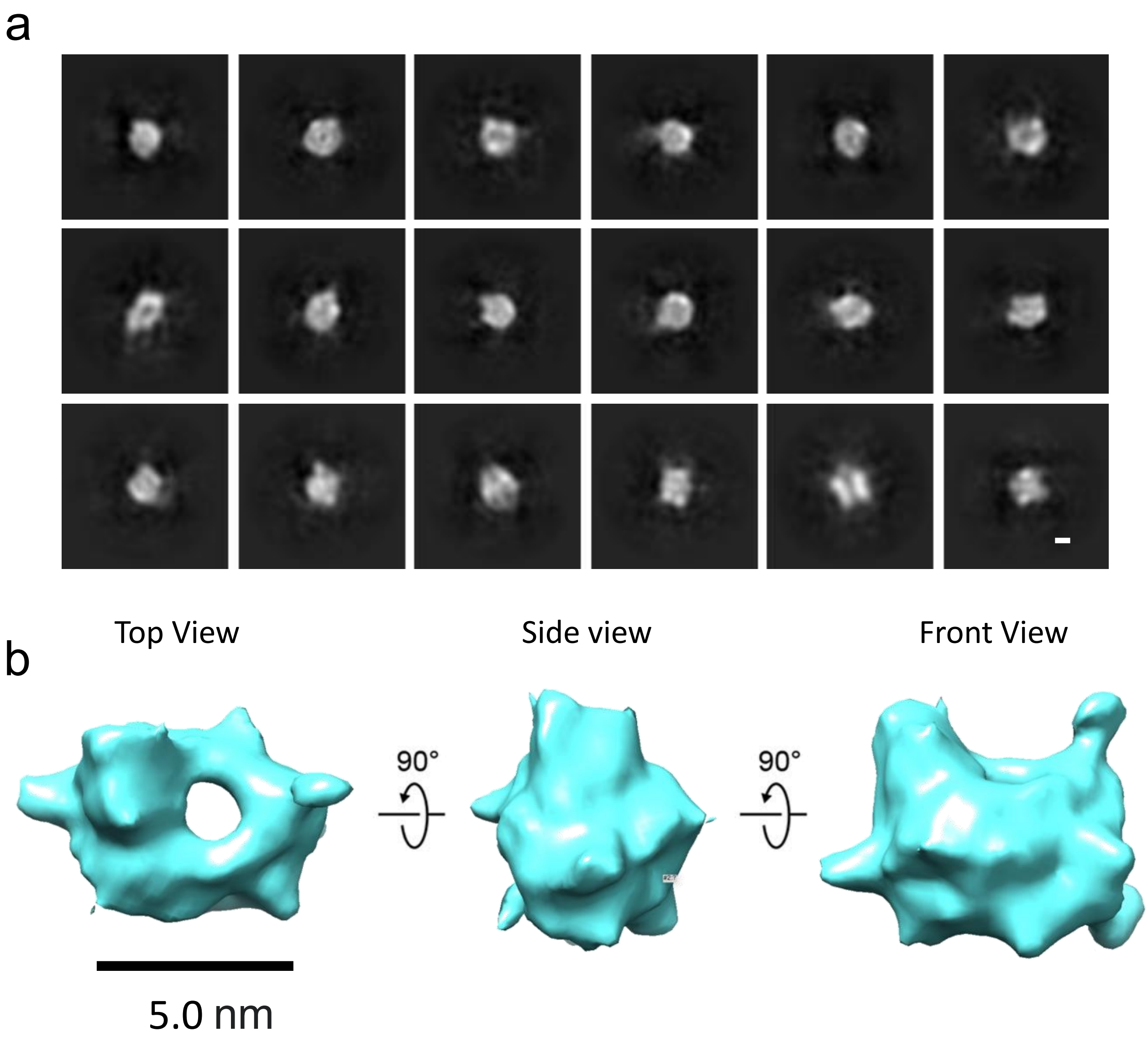

Supplemental Figure S9. Cryo-EM 3D reconstruction of annular assemblies. 2D averages of 144,593 particles (a) and 3D reconstruction (b). Annular structure with an internal pore, through the middle of the structure. Dimensions of 3D: 8.6 nm; 7.4 nm; 6.4 nm . Scale bar: 5 nm for 3D structures, 2 nm for 2D averages.

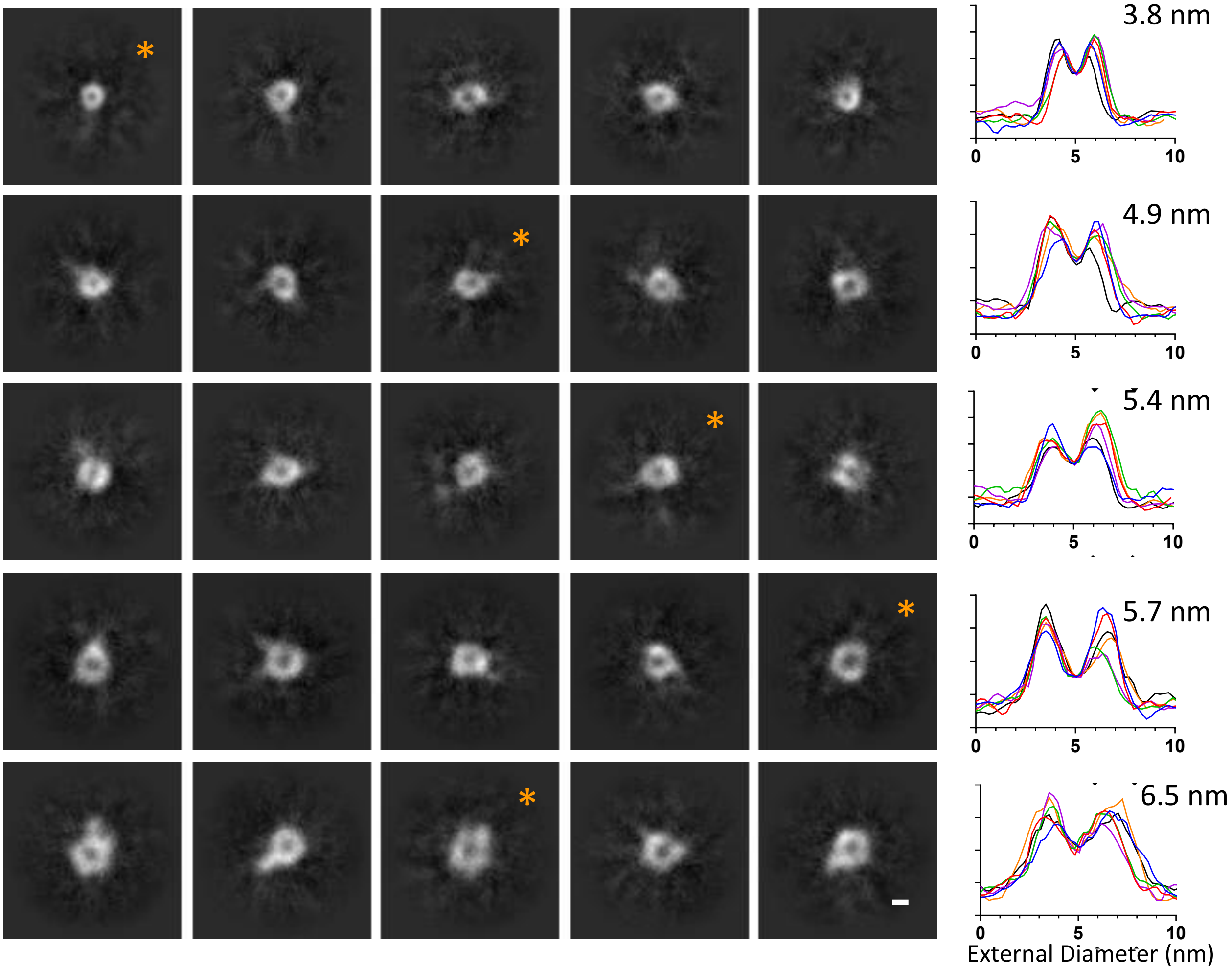

Supplemental Figure S10. Cryo-EM Annular oligomers top view. 2D class averages for top (and bottom) views of annular oligomers, ranked by size, with the smaller rings on the top rows. Each class average contains ~400 particles; 10,000 particles in total. Scale bar 2.0 nm. To the right shows density profiles for representative annular oligomers top view, labeled by *orange, one from each row. Six profiles for angle 0, 30, 60, 90, 120, and 150 degree in colour red, orange, green, blue, black and purple. The external diameter measure from baseline of the plot profiles (row 1 to 5): 3.8, 4.9, 5.4, 5.7 and 6.5 nm.

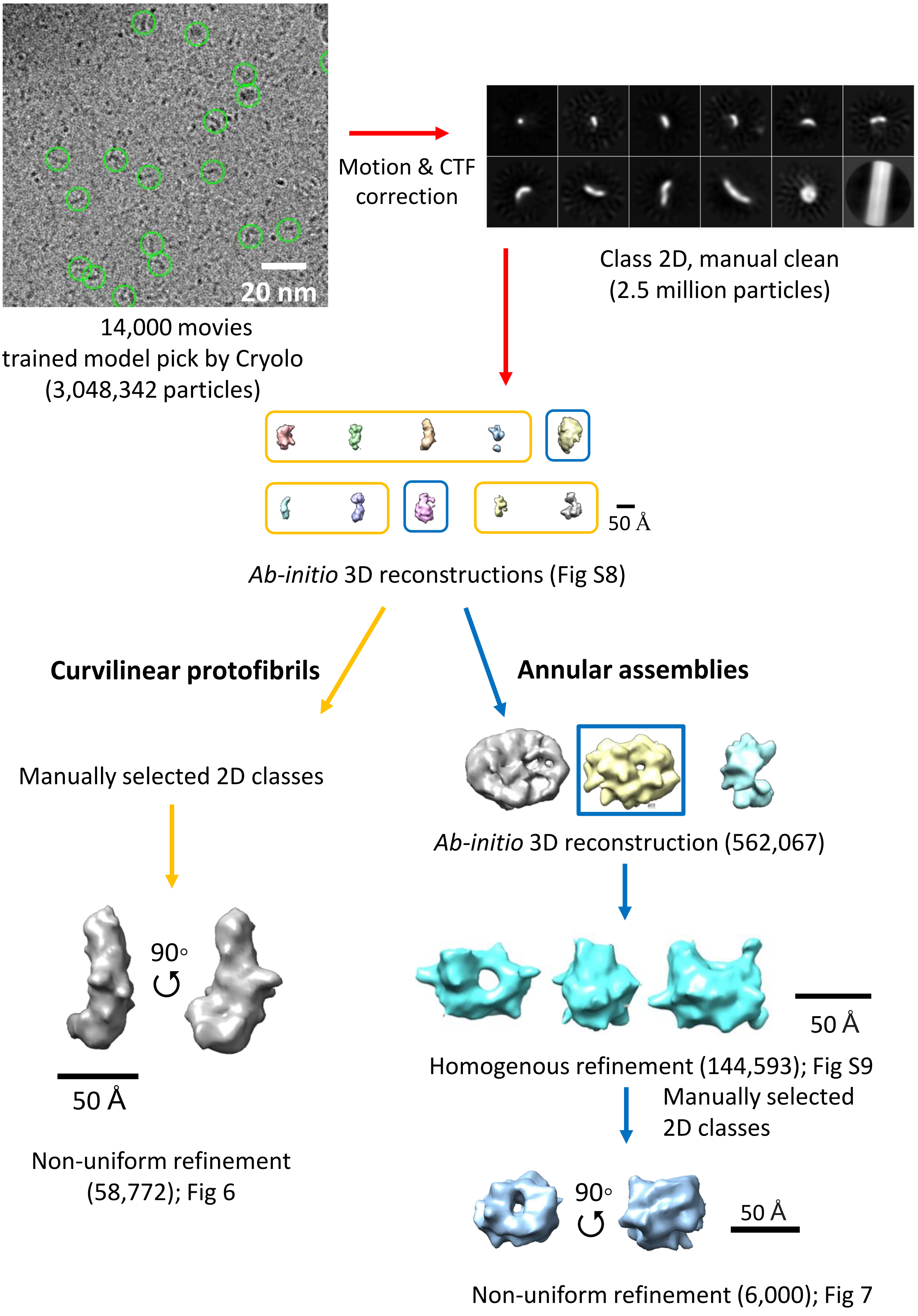

Supplemental Figure S11: Workflow, Cryo-EM, Single Particle Analysis

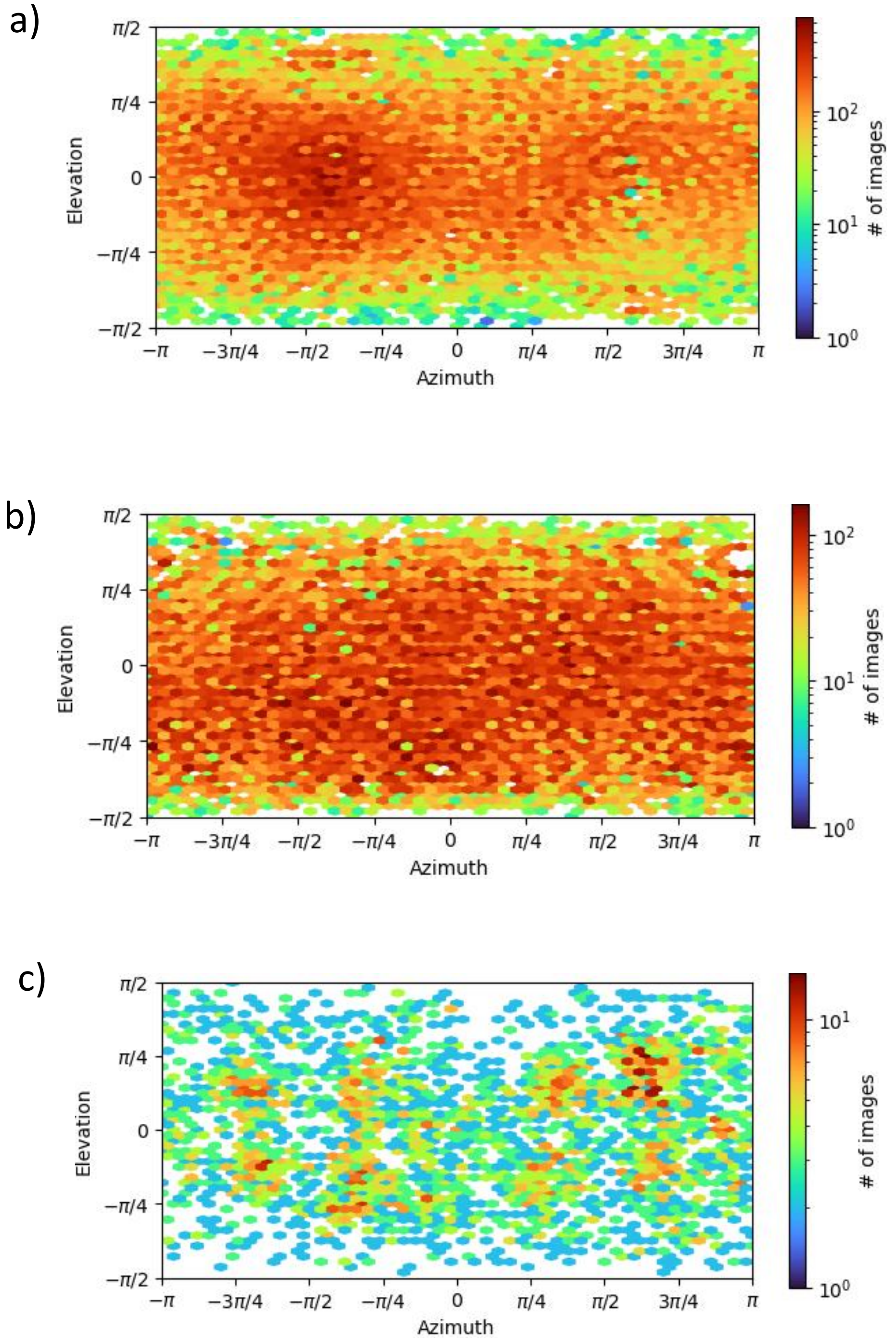

Supplemental Figure S12. Angular distribution for 3D reconstruction of Curvilinear protofibrils and Annular assemblies. (a) Structure Curvilinear protofibrils shown in Figure 6, 59 k particles. **(b)** Annular Structure shown in Figure S9, 144 k particles. **(c)** Refined annular structure shown in Figure 7, 6.5 k particles. The angular distribution does not show any marked orientational bias within the structures.

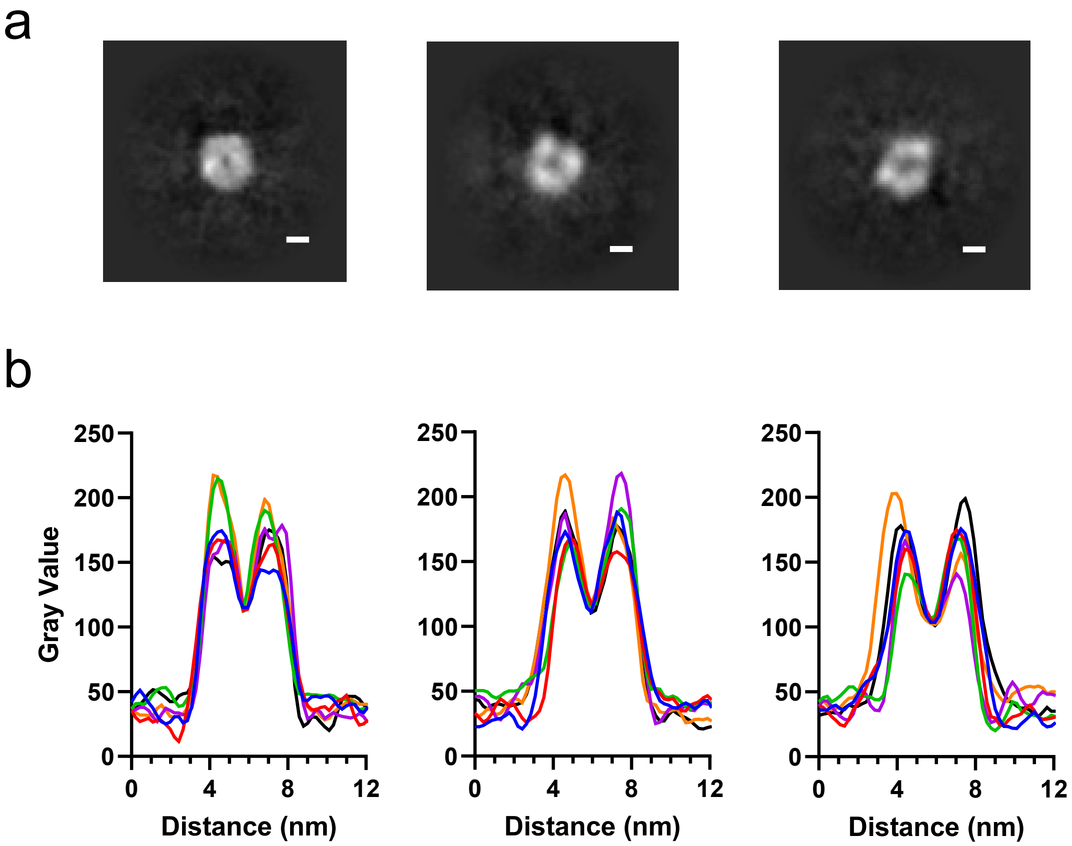

Supplemental Figure S13. Cryo-EM 2D class averages and density profiles of annular particles. a) The representative class averages of top view of annular oligomers. b) density profiles across the annular oligomers top view. Six profiles for angle 0, 30, 60, 90, 120, and 150 degree in colour red, orange, green, blue, black and purple. Scale bar : 2.0 nm.

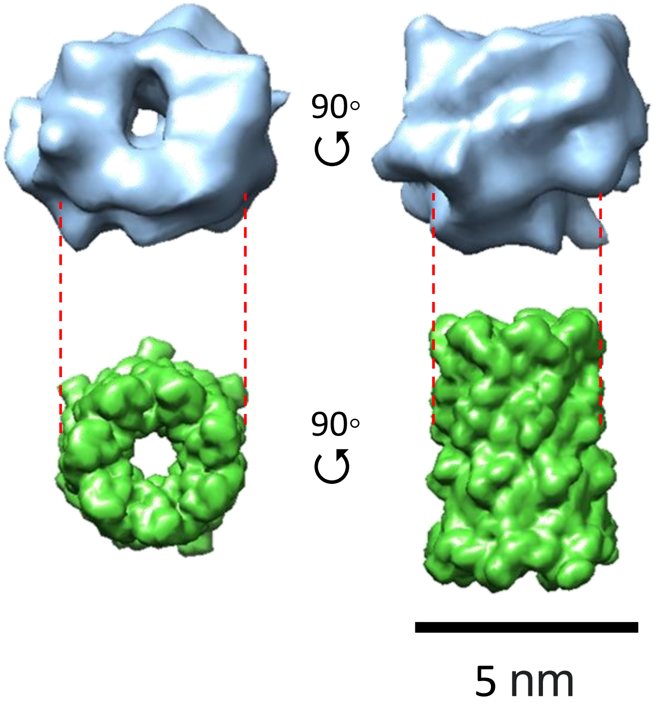

Supplemental Figure S14. Comparison of annular oligomer of Aβ_{42} wild-type (Blue) with a Aβ42 fixed as a heptameric β-hairpin structure with α-hemolysin as a scaffold (Green) pdb=7O1Q. The annular oligomers of wild-type Aβ_{42} is wider but not as longer as the β-hairpin contain seven Aβ_{42} molecules. The density for the scaffold dependent β-hairpin contains all 42 amino acid, which may not be the case for the cryo-EM image of Aβ_{42} annular oligomers. Scale bar: 5 nm.

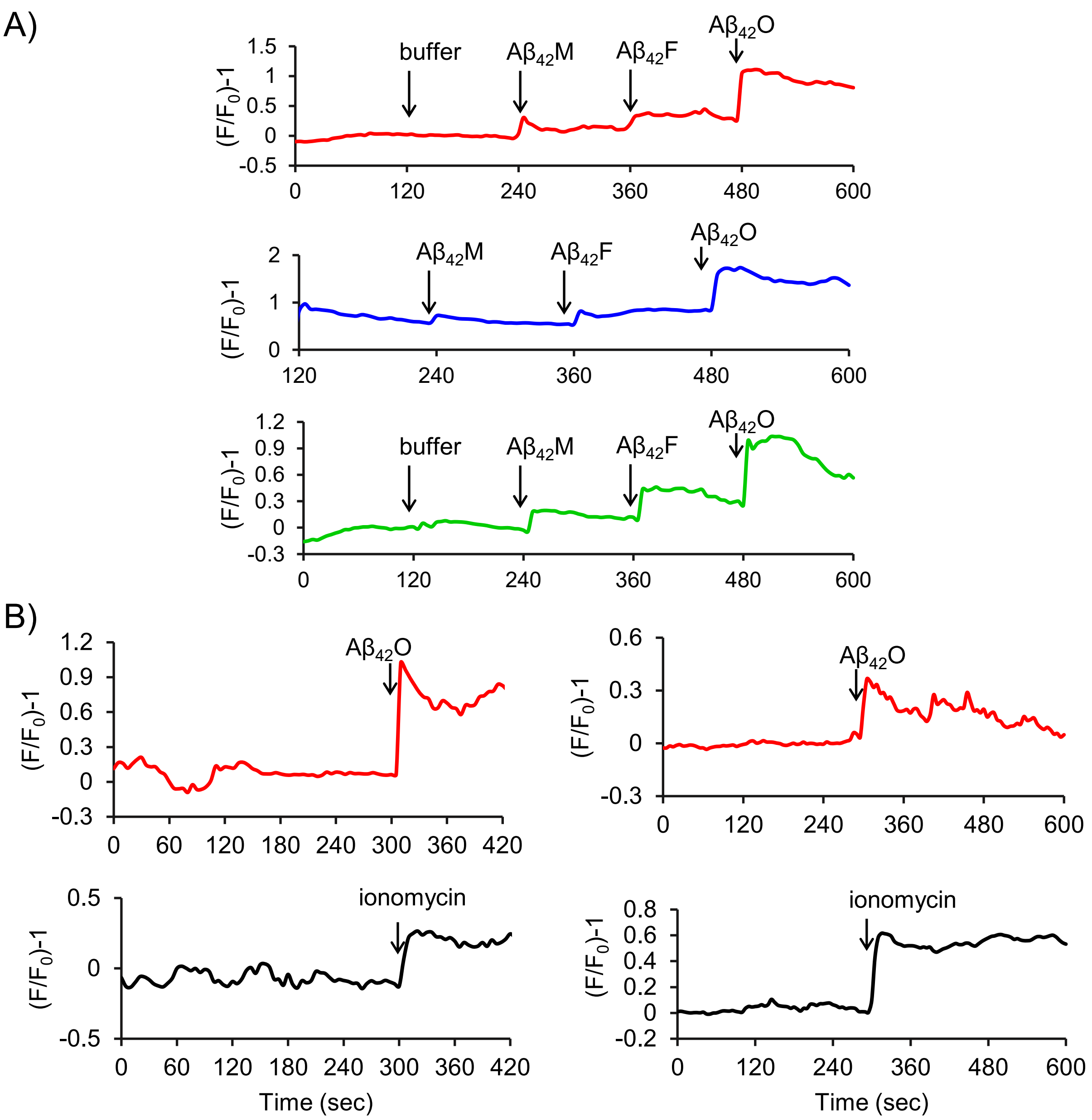

Supplemental Figure S15: Cellular Ca^{2+} detection in HEK293 cells by Floro-4. Comparison of impact of just buffer, $A\beta_{42}$ monomer, fibril and finally oligomers (A). Comparison of impact of $A\beta_{42}$ oligomer with ionomycin. Final $A\beta_{42}$ concentration 5 μM, pH 7.4.

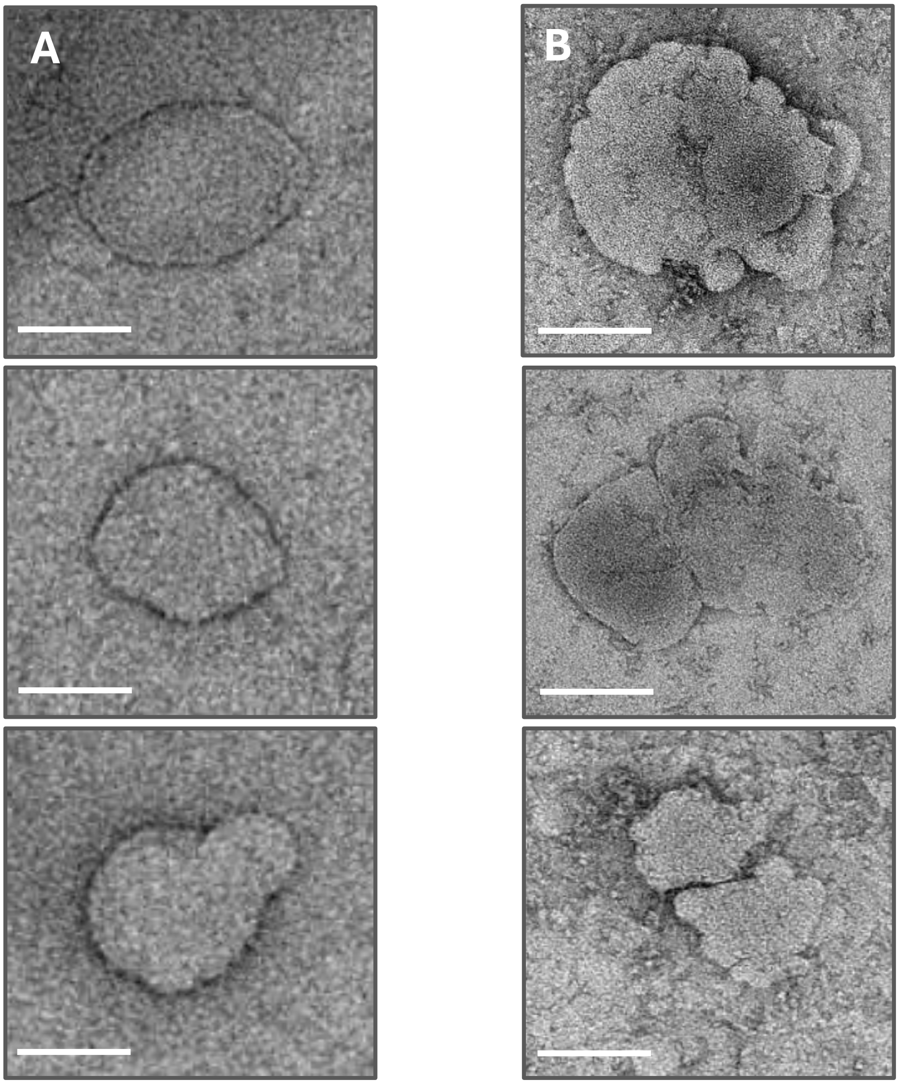

Supplemental Figure S16: TEM images of lipid vesicles without (A) and with the presence of Aβ_{42} pre-fibrillar assemblies (B). Aβ_{42} oligomers and curvilinear protofibrils incubated with LUVs for just 30 s. Aβ_{42} prefibrillar assembles were obtained at the end of the lag-phase during fibril formation. Widespread distortions in the appearance of the vesicles are apparent in the presence of oligomers and curvilinear protofibrils in less than 1 min after addition of Aβ. LUVs contain PC:cholesterol:GM1 (68:30:2 by weight). Images are negatively stained with uranyl acetate. Scale bar 50 nm.

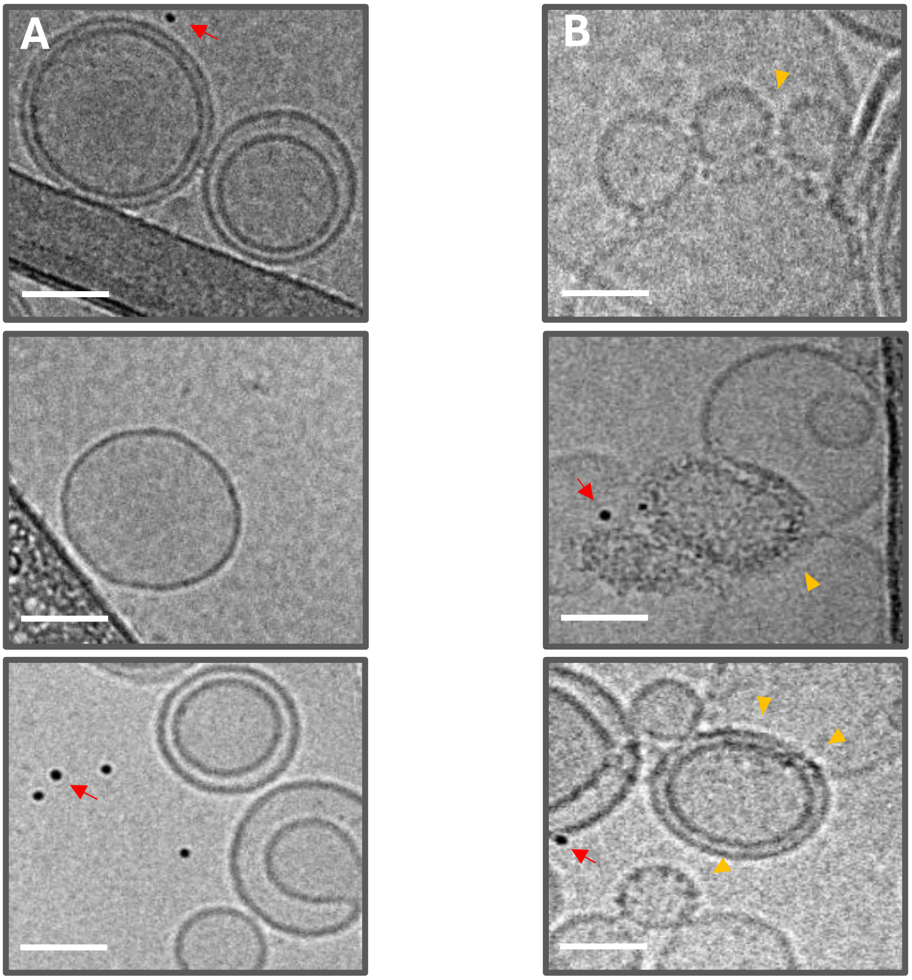

Supplemental Figure S17: Cryo-EM images of lipid vesicles without (A) and in the presence of Aβ_{42} pre-fibrillar assemblies (B). Aβ_{42} oligomers and curvilinear protofibrils incubated with LUVs for just 30 s. Within less than a minute the vesicle surface shows Aβ assemblies in the lipid bilayer (yellow triangle). LUVs (0.5 mg mL^{-1}) contain PC:cholesterol:GM1 (68:30:2) by weight. Scale bar 100 nm, Magnification 50,000x. Red arrow represents a gold fiducial marker.

	Conductance (pS)			
Patch no.	20%	50%	80%	Difference (20% and 80%)
1	87	217	348	261
2	94	235	376	282
3	102	255	408	306
4	114	284	454	341
**5**	**130**	**320**	**510**	**380**
6	146	364	582	437
7	153	382	611	458
8	167	417	667	500
9	226	564	902	677
**Average**	135	337	539	405
**SD**	44	109	174	131

Table S1: Conductance of Aβ_{42} channels recorded from nine individual patches. Taken from Figure 6B. The range of conductance's for each of the nine channels is shown as 20%, 50%, and 80% of the maximum conductance value. The channel with the median conductance is highlighted in bold. The mean and standard deviation are displayed at the bottom of the table for reference.

Conductance (pS)	Implied diameter (nm)	
	5.4 nm pore length	7.0 nm pore length
200	1.1	1.3
250	1.3	1.4
300	1.4	1.6
350	1.5	1.7
400	1.7	1.9
450	1.8	2.0
500	1.9	2.1
550	2.0	2.2
600	2.1	2.3
650	2.2	2.4
700	2.3	2.5

Table S2 **Predicted Inner Diameter of channel calculated from Conductance.** The implied diameter (nm) has been calculated for conductance's between 200 and 700 pS, with two possible channel pore lengths of 5.4 and 7.0 nm.

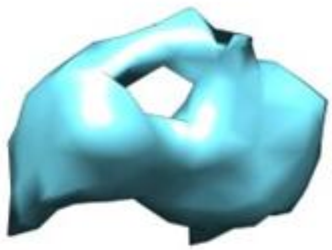

Movie S1: Aβ_{42} annular assemblies imaged in 3D by Cryo-ET, 360° view. Still from the movie is shown above. Surface rendered 3D image. External diameter of ring *ca.* 7 nm, internal length of channel *ca.* 3 nm.
